# Modeling of annexin A2—Membrane interactions by molecular dynamics simulations

**DOI:** 10.1371/journal.pone.0185440

**Published:** 2017-09-22

**Authors:** Davit Hakobyan, Volker Gerke, Andreas Heuer

**Affiliations:** 1 Institute of Physical Chemistry, University of Muenster, Muenster, Germany; 2 Center for Multiscale Theory and Computation (CMTC), University of Muenster, Muenster, Germany; 3 Institute of Medical Biochemistry, Center of Molecular Biology of Inflammation (ZMBE), University of Muenster, Muenster, Germany; Russian Academy of Medical Sciences, RUSSIAN FEDERATION

## Abstract

The annexins are a family of Ca^2+^-regulated phospholipid binding proteins that are involved in membrane domain organization and membrane trafficking. Although they are widely studied and crystal structures are available for several soluble annexins their mode of membrane association has never been studied at the molecular level. Here we obtained molecular information on the annexin-membrane interaction that could serve as paradigm for the peripheral membrane association of cytosolic proteins by Molecular Dynamics simulations. We analyzed systems containing the monomeric annexin A2 (AnxA2), a membrane with negatively charged phosphatidylserine (POPS) lipids as well as Ca^2+^ ions. On the atomic level we identify the AnxA2 orientations and the respective residues which display the strongest interaction with Ca^2+^ ions and the membrane. The simulation results fully agree with earlier experimental findings concerning the positioning of bound Ca^2+^ ions. Furthermore, we identify for the first time a significant interaction between lysine residues of the protein and POPS lipids that occurs independently of Ca^2+^ suggesting that AnxA2-membrane interactions can also occur in a low Ca^2+^ environment. Finally, by varying Ca^2+^ concentrations and lipid composition in our simulations we observe a calcium-induced negative curvature of the membrane as well as an AnxA2-induced lipid ordering.

## Introduction

Annexins are peripheral membrane-binding proteins involved in the regulation of membrane organization and membrane traffic [1, 2]. More than 100 Annexins have been identified in different species and twelve proteins referred to as Annexin A1-13 (A12 gene is unassigned) have been identified in humans. Annexins reversibly associate with the cytosolic surface of cellular membranes in a Ca^2+^-dependent manner and are involved in the control of membrane organization, membrane-cytoskeleton contacts and membrane trafficking events (for reviews see [1–3]). Annexin A2 (AnxA2) is a member of the family that serves important functions in organizing membrane microdomains and regulating Ca^2+^-evoked exocytosis in non-neuronal cells. Moreover, the protein has been implicated in early endocytic events and the resealing of plasma membrane wounds [1, 2, 4, 5]. AnxA2 can exist in cells as a monomer and as a heterotetramer consisting of two annexin A2 molecules and one p11 dimer. S100A10 itself is a member of the Ca^2+^-binding S100 protein family [6]. Heterotetrameric AnxA2-S100A10 is mainly localized at the plasma membrane [7–10], while the monomer is mainly cytosolic but can also associate with membranes under certain conditions. The N-terminal tail of AnxA2, which precedes the annexin core and varies in length and sequence in comparison to other annexins, mediates the interaction with S100A10 and thereby regulates AnxA2 properties [11]. It is suggested that regulation of AnxA2 via its N-terminal S100A10 binding could increase the efficiency of Ca^2+^-induced membrane attraction and could stabilize membrane bridges formed by two AnxA2 subunits binding to two membranes, i.e. the crosslinking of two membrane surfaces by the AnxA2-S100A10 heterotetramer [12]. The N-terminal domain is easily cleaved by mild proteolysis, leaving a 33 kDa protein core which comprises, like in all other annexins of the 35 to 40 kDa class, four conserved repeats each of which is composed of five α helices [2], and spans 70–80 amino acids. The core domain harbors the binding sites for the common annexin ligands, phospholipid and Ca^2+^ [13–15].

The influence of calcium ions on membranes is of key scientific interest. A recent study showed that lipid bilayers strongly absorb calcium ions, leading to a compression of bilayers. This suggests that the inner leaflet of a cellular membrane can act as a calcium buffer and modulate calcium diffusivity in calcium signaling microdomains [16]. In case of neutral bilayers with zwitterionic 1-palmitoyl-2-oleoyl-sn-glycero-3-phospho-choline (POPC) and 1-palmitoyl-2-oleoyl-sn-glycero-3-phosphoethanolamine (POPE) lipids the calcium Ca^2+^ ions were suggested to prefer binding to positively bended bilayer regions [17]. For DPPC lipids it was observed that both Ca^2+^ and Zn^2+^ ions induce a thickening of the bilayer while further increase of ion concentrations results in bilayer thinning in case of Ca^2+^ and to thickening saturation in case of Zn^2+^ [18]. For bilayers with negatively charged 1-palmitoyl-2-oleoyl-sn-glycero-3-phospho-L-serine (POPS) and phosphatidylinositol (4, 5)-biphosphate (PI(4,5)P_2_) lipids calcium ions were suggested to induce negative curvature at places where Ca^2+^ ions interact with negatively charged lipids [19]. While the reported induction of negative curvature is weak the authors further suggest that local Ca^2+^ signals could act in concert with curvature generating proteins to trigger membrane deformations. This hypothesis may very well apply to AnxA2 and Ca^2+^ mediated bilayer interaction.

**Fig 1:**
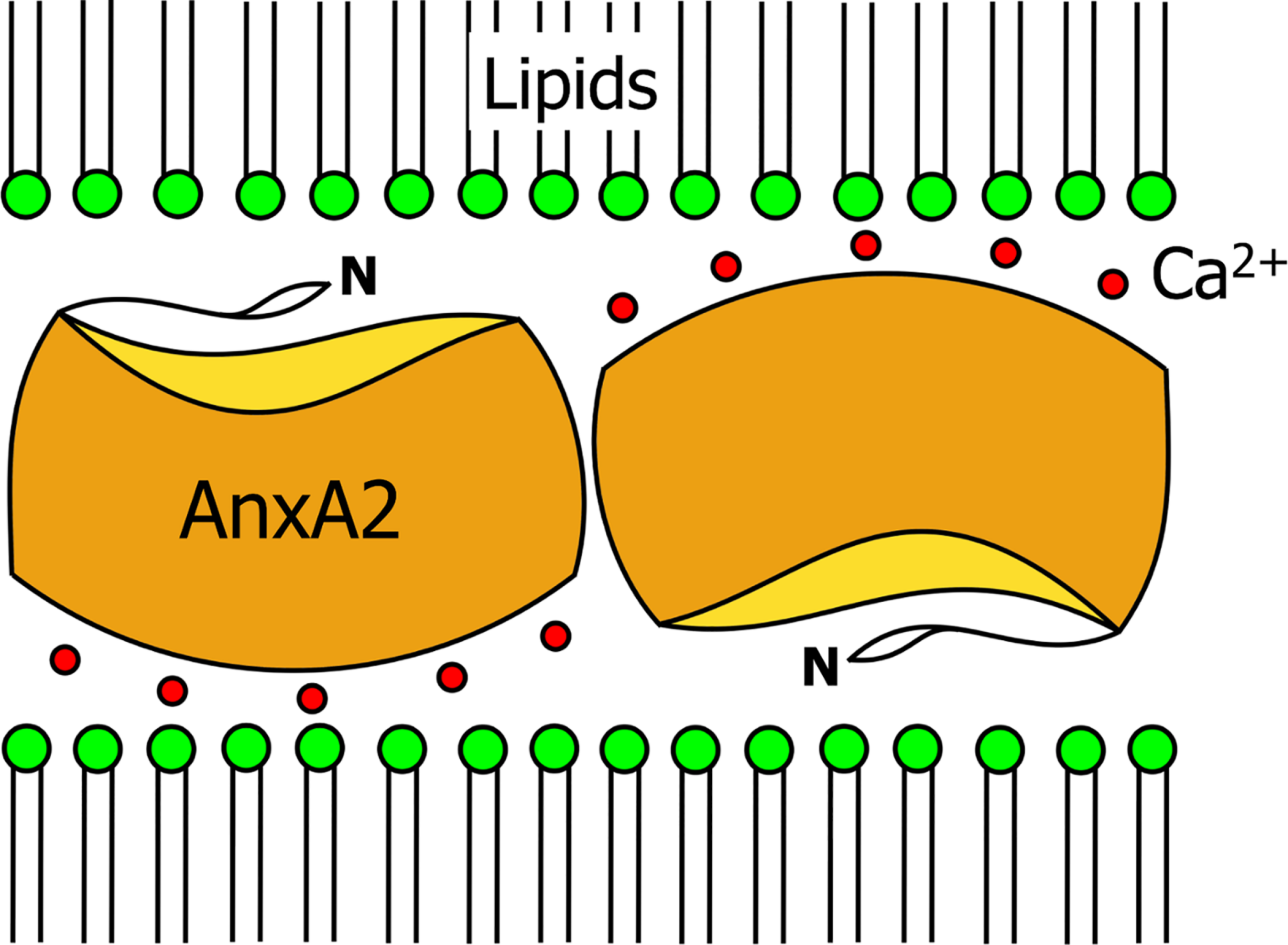
Sketch of a Ca^2+^-mediated AnxA2 dimer configuration as suggested in [25].

A large number of experimental studies was devoted to analysis of AnxA2 –membrane binding. Particularly, it has been shown that AnxA2, like most other annexins specifically binds to negatively charged phospholipids in the presence of Ca^2+^ and has a high affinity for PI(4,5)P_2_ and phosphatidylserine (PS). This corresponds to a recruitment of the AnxA2-p11 complex to PI(4,5)P_2_-rich sites of membrane-associated actin assembly that is observed in cells [20]. It was proposed that the heterotetrameric AnxA2 forms two-dimensional protein clusters and recruits negatively charged PS lipids (e.g. POPS or 1,2-dipalmitoyl-sn-glycero-3-phosphoserine [DPPS]) to underneath these clusters [21]. Monomeric and heterotetrameric AnxA2 were found to require different Ca^2+^ concentrations for membrane POPS binding (differing by up to 3 orders of magnitude) [22–24]. Moreover, Ca^2+^ ions were suggested to cluster adjacent monomeric AnxA2 in an „up-side-down”fashion, thus presumably enabling the molecules to bind to two membranes [25]. Fig 1 shows this postulated dimeric “up-side-down” configuration. Here the calcium ions are found on both sides of the AnxA2 dimer enabling it to bind to two membranes simultaneously [25]. The interaction between the monomers of the AnxA2 dimer is stabilized not only by the salt bridges but also by the presence of Ca^2+^ at the inter-protein junction region. Interestingly, in the presence of calcium AnxA2 can, possibly, also form “tail-to-tail” or “head-to-head” configurations where one AnxA2 is sitting on top of the other thus forming an AnxA2 double layer connecting two membranes [26, 27].

The Ca^2+^ mediated AnxA2-membrane binding is interesting both in terms of membrane reorganization as well as of changes of AnxA2 binding properties. Different forms of AnxA2 (core, monomer, heterotetramer) can affect lipid segregation in membranes composed of POPC, 1,2-dioleoyl-sn-glycero-3-phosphocholine (DOPC), PI(4,5)P_2_, POPS, and cholesterol (CHOL) [28]. Here it was suggested that AnxA2 primarily and directly interacts with PI(4,5)P_2_ in a Ca^2+^-dependent manner and clusters this lipid into larger domains. On the other hand binding of Ca^2+^ to annexins in the absence of membranes induces a conformational change of the protein [24, 29–33]. The major difference between the membrane-free and membrane-bound forms of the Ca^2+^—AnxA2 complex is the Ca^2+^ sensitivity [27]. Therefore, it is of major interest to study the Ca^2+^ mediated AnxA2 –membrane interaction on the microscopic level which, however, is not yet possible with experimental techniques. Due to the absence of membrane structures in the protein crystals, the experimental observations of Ca^2+^-mediated AnxA2 configurations interacting with membrane remain mostly indirect.

Some computer simulation studies were conducted previously to investigate annexin–membrane interaction. For example, MD simulations limited to 7 ns were performed to study the conformation changes of the Ca^2+^-bound Annexin A1 as compared to the Ca^2+^-free protein without the presence of a membrane [34]. Another two MD studies with simulation times less than 60 ns were devoted to studying the influence of the N-terminal part of the molecule to the binding affinity of preconfigured Annexin A1 to the membrane [35, 36]. In the case of annexin A5 another force field was suggested [37]. The Ca^2+^-dependent annexin A5—membrane interactions were also investigated to study interaction specificities as well as the influence of annexin A5 on the properties of the membrane, i.e. lipid order parameters, membrane bending and formation of H-bonds [38]. Moreover, MD simulations support the formation of supramolecular assemblies of annexin A5 in Ca^2+^-dependent membrane binding experiments [39]. Monte Carlo simulations based on experimental data were also performed to model annexin A5 –membrane interaction and suggested that the protein–PS interaction together with a protein–protein interaction provide a driving force for the formation of large PS-rich domains and that the presence of cholesterol may provide a further contribution to the clustering of such PS domains [40]. In the case of annexin B12, MD simulations in combination with NMR studies were employed to investigate water diffusion and retardation effects as a function of distance from annexin B12 and the membrane [41].

However, in all the referenced MD studies the annexin had a predefined orientation relative to the membrane surface thus introducing a certain bias towards the mode of membrane interaction. Furthermore, systematic data on the interaction of plasma membrane-associated AnxA2 with the membrane on the atomistic scale are not available. This would be of significant general interest as it could represent a paradigm for reversible phospholipid interactions of peripheral membrane-binding proteins.

Therefore, we addressed this question by MD simulations of monomeric AnxA2 in Ca^2+^-containing membrane systems.

A large number of protein-protein and protein-ligand docking techniques and tools do exist [42–44]. For example in one of the recent studies around 382 billion configurations were tested for docking cytochrome c_2_ and bc_1_ complex using the rigid-body docking program DOT 2.0 [45]. We refrained from using this methodology for the following reasons. First, the topology of one of the constituents (membrane) is very simple so that only the protein needs to be rotated.Second, due to the complex binding properties, involving the appropriate localization of Ca^2+^ ions, possible conformational changes of AnxA2 as well as reorganization of the local membrane composition, each attempt requires a long additional simulation in the range of μs. Thus, only a somewhat limited number of attempts can be used. Third, as explicitly shown in our work these simulation times are long enough so that even transitions between different initial orientations are possible. This strongly suggest, that we have indeed managed to cover the relevant initial orientations. However, the docking approaches could be rather efficient for the investigation of more complex systems such as aggregations of multiple AnxA2.

In the first simulation setup we keep the membrane and Ca^2+^ composition the same and vary the orientation of AnxA2 in respect to the membrane. In these simulations AnxA2 was positioned above a POPC:DOPC:POPS:CHOL (0.38:0.19:0.24:0.19) membrane in 18 different orientations. In this way we systematically and efficiently assessed which surface residues of AnxA2 can favorably interact with the membrane. The Ca^2+^ ions were positioned between AnxA2 and the membrane to, possibly, mediate AnxA2 –membrane binding. In the second setup we keep AnxA2 orientation (identified in the first setup as favorable) the same and change the composition of the membrane (by adding PI(4,5)P_2_ lipids) as well as the concentration of Ca^2+^. The influence of AnxA2 and Ca^2+^ ions on membrane curvature and ordering is investigated.

The aims of this MD work were to i) determine the favorable AnxA2 –membrane binding configurations, if any, ii) quantify the influence of Ca^2+^ ions on AnxA2 –membrane total enthalpy, iii) compare the MD results with experimental findings, iv) investigate possible energetic differences of AnxA2 interactions with different types of lipids, and v) identify the protein residues which are responsible for phospholipid binding vi) determine the effect of AnxA2/membrane/Ca^2+^ interactions on membrane bending and ordering.

## Results

### Binding enthalpies

**Fig 2:**
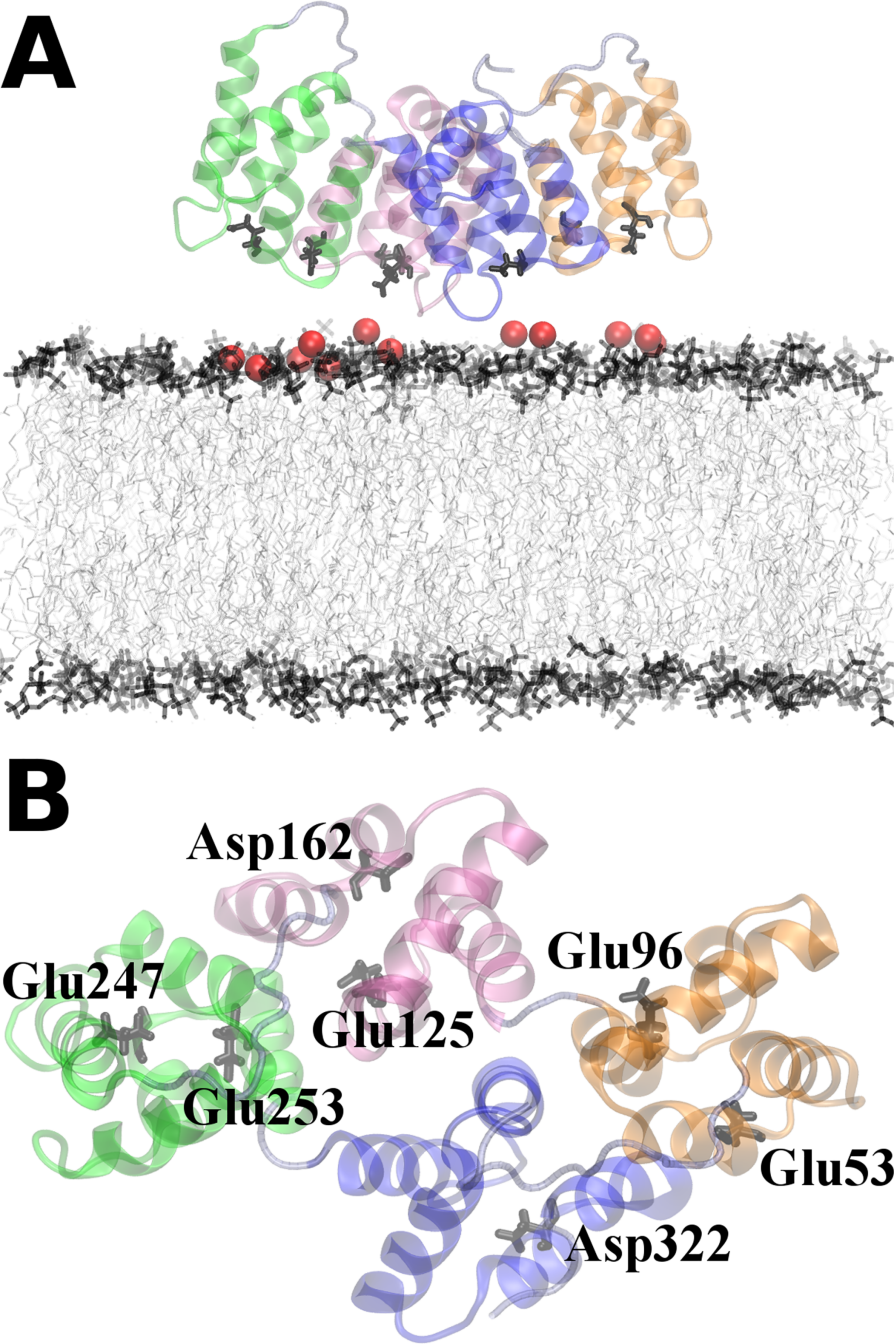
The initial system. The side view and the view from top of the initial system are shown. This system corresponds to the O2 configuration. The four AnxA2 repeats are shown in different colors: repeats 1 (Ala35 –Lys104), 2 (Ala107 –Lys176), 3 (Asp187 –Ile263) and 4 (Lys266 –Cys335) are colored in orange, mauve, green and blue, respectively. The red spheres located between AnxA2 and the membrane are calcium ions. The membrane lipids are shown in gray.

The initial structure from which the 18 orientations were generated is shown in Fig 2.

**Fig 3:**
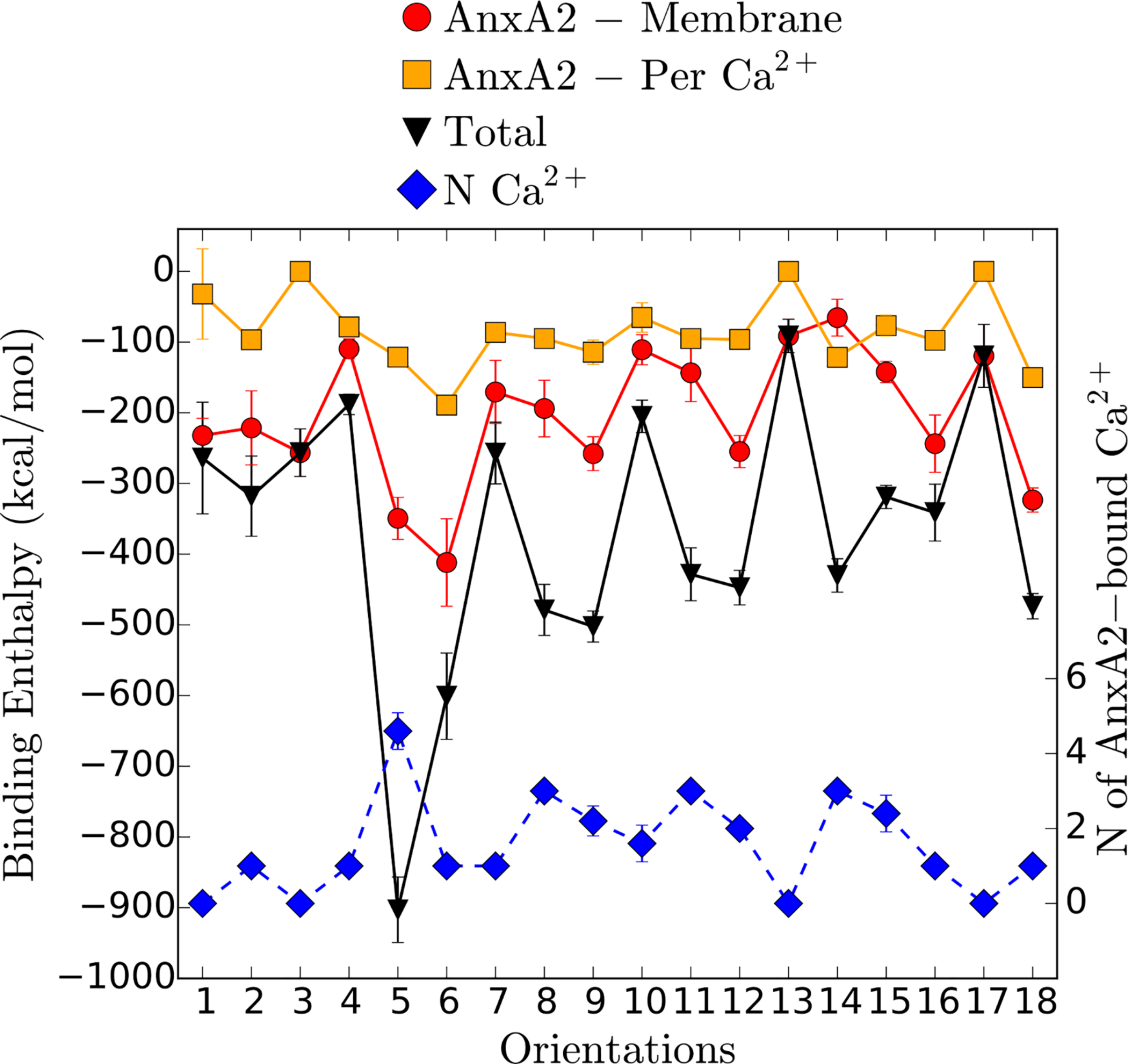
Binding enthalpies and number of Ca^2+^ ions around AnxA2. The energy values (including standard deviations) and the number of neighbors are the averages determined for the last 50 ns of the MD simulation. Criteria for selection of the Ca^2+^ are given in the main text.

A total of 18 orientations was obtained by a stepwise rotation of the folded AnxA2 around the axes shown (the snapshots of initial systems are shown in S1 Fig). First we calculate the different binding enthalpies, characterizing the interaction of AnxA2 with the membrane and the calcium ions, respectively, for the different initial orientations (Fig 3). Only those Ca^2+^ ions are considered which, with respect to AnxA2 and the membrane have an interaction energy larger than -1 kcal/mol and a distance of less than 10 *Å*. The exact choice of these criteria, however, has only a minor influence on the final result. The total binding enthalpy for AnxA2 is the sum of AnxA2 –membrane and AnxA2 –Ca^2+^ enthalpies. We exclude the membrane–Ca^2+^ enthalpy because it strongly depends on the PS lipid localization and also because our primary interest is the AnxA2 –Ca^2+^/membrane interaction.

One of the immediate observations in Fig 3 is the rather strong enthalpic interaction of AnxA2 and the membrane-Ca^2+^ complex for orientation O5. Although the AnxA2 –membrane enthalpy in O5 is one of the strongest (-350 kcal/mol), the largest difference as compared to other orientations results from the AnxA2 –Ca^2+^ interactions (additional -550 kcal/mol). It should be noted that the number of Ca^2+^ ions presented in Fig 3 is time averaged and limited by the threshold of -1 kcal/mol. While the number of Ca^2+^ in Fig 3 for O5 is close to 5 a closer inspection of AnxA2-Ca^2+^ interaction energies (data not shown) reveals six, strongly interacting and membrane-exposed, Ca^2+^ ions. The discrepancy is due to a very weak interaction of one of those six Ca^2+^ ions (bound to Glu156 residue) with the membrane. We consider this as a transient effect related to the absence of nearby negatively charged POPS lipids and, therefore, in further discussion of O5 configuration we consider all six Ca^2+^ ions.

**Fig 4:**
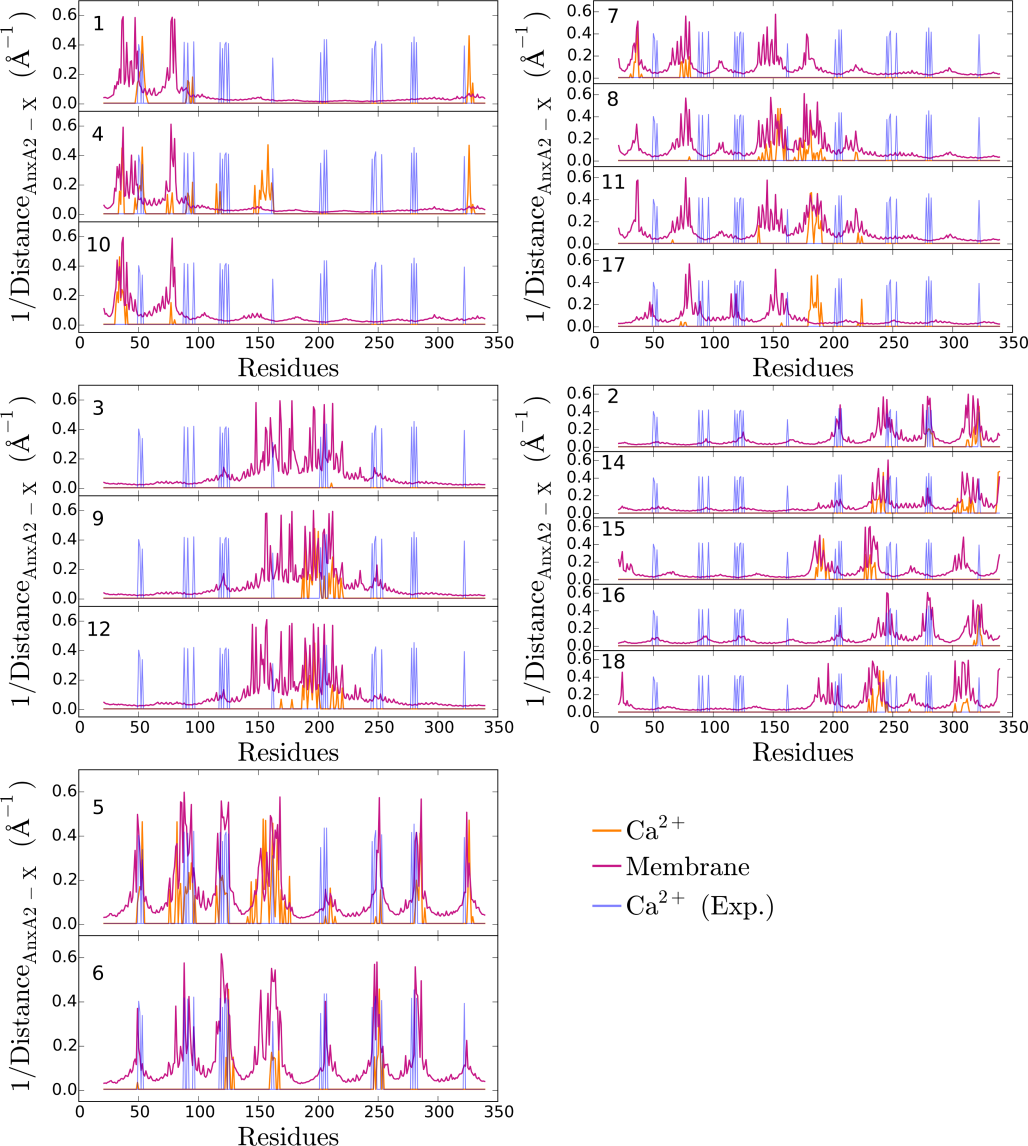
Structural properties of the different orientations with respect to the membrane and the Ca^2+^ ions. To identify the residues which are positioned most closely to either the membrane lipids or the Ca^2+^ ions, we show the inverse of their minimal distance. The data presented are the average values of the last 50 ns of the simulations. The blue peaks are the experimentally determined inverse distances between AnxA2 and Ca^2+^ ions found in the crystal structure of AnxA2 [25]. The number in each subplot represents the orientation. Orientations have been grouped according to their binding pattern.

Next, we extracted from our simulations the inverse minimal distance of AnxA2 to either the membrane or the Ca^2+^ ions for 17 of the 18 orientations (Fig 4). For comparison, the corresponding values for the AnxA2—Ca^2+^ pairs in the experimentally determined crystal structure are also shown [25].

**Fig 5:**
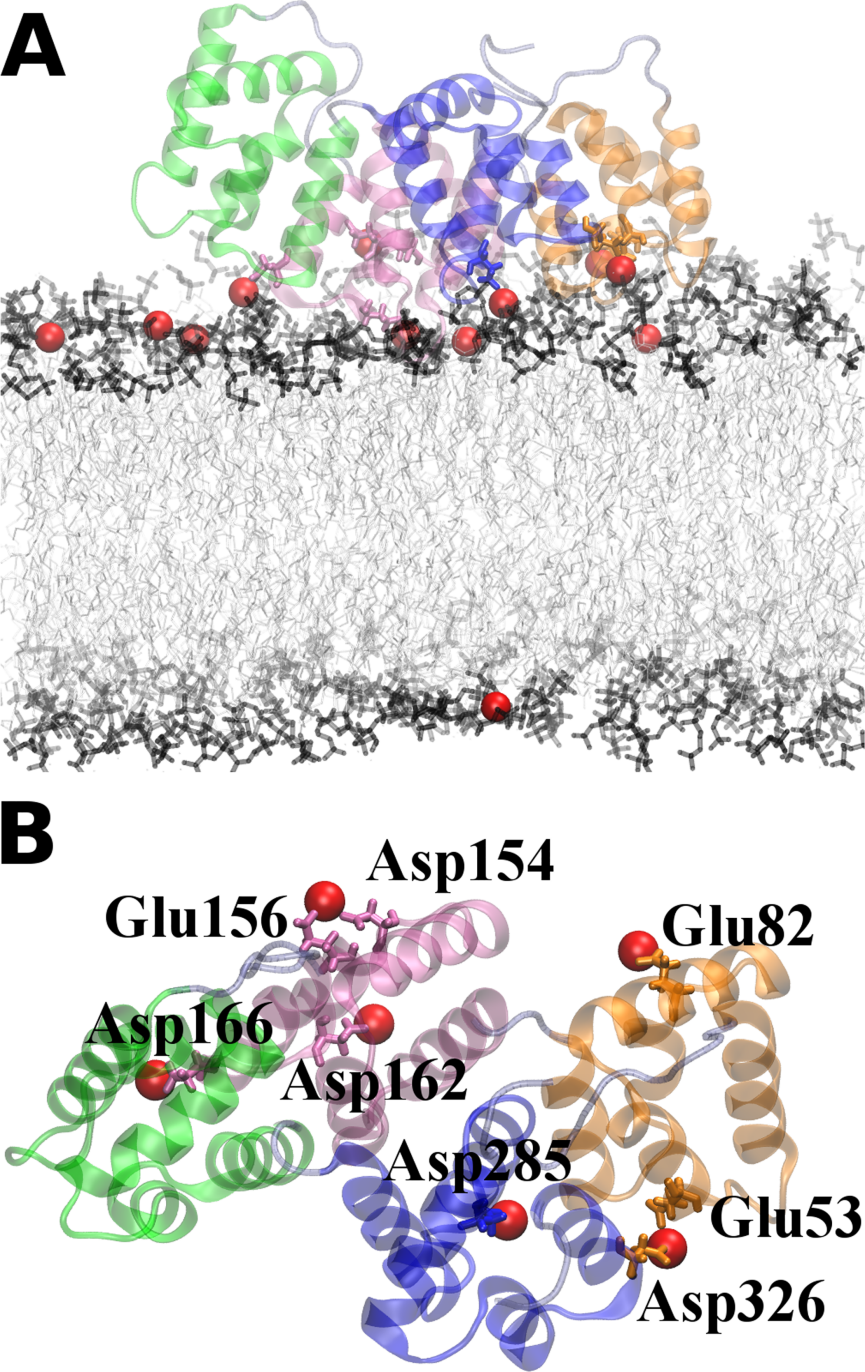
Snapshots of the system O5 after 400 ns. The repeats 1 (Ala35 –Lys104), 2 (Ala107 –Lys176), 3 (Asp187 –Ile263) and 4 (Lys266 –Cys335) of AnxA2 are colored in orange, mauve, green and blue, respectively. The relevant Ca^2+^ ions are shown as red spheres. Amino acid residues participating in Ca^2+^ biding are highlighted. (A) Profile view and (B) view from top. Water, NaCl as well as the membrane molecules are not shown.

Remarkably, only O5 (and to a lesser extent O6) showed very good agreement with the crystal structure data with respect to the protein-Ca^2+^ interactions. This is the same orientation that showed the strongest interaction with the membrane-Ca^2+^ complex in the enthalpy calculations (Fig 3). In total, six binding sites of Ca^2+^ are identified in O5. These include the following residues: Glu53 and Asp326 (repeat 1 and 4), Glu82 (repeat 1), Asp154 and Glu156 (repeat 2), Asp162 (repeat 2), Asp166 (repeat 2), Asp285 (repeat 4). This shows that the repeat 2 –membrane binding should play a major role in stabilizing the Ca^2+^ -mediated membrane interaction in the favorable O5 configuration. The additional Ca^2+^ binding site found in the crystal structure around the residue Asp200 is positioned in the configuration O5 rather distant from the membrane and should not be considered a AnxA2 –membrane binding site. Rather this site could be involved in stabilizing a dimeric AnxA2 complex [25]. Fig 5 shows the final structure (after 400 ns) of AnxA2 together with bound Ca^2+^ ions in the O5 configuration.

Interestingly, for O6, which closely resembles O5, another binding site is identified which is missing in O5 (i.e. Glu125 and Asp251 which involve repeats 2 and 3) and was found when inspecting the vicinity during the MD simulations. Most likely, longer simulations allowing more thermal fluctuations of the Ca^2+^ positions would have revealed this binding site in O5 as well. In summary, our numerical observations show excellent agreement of the binding sites in the O5 orientation with the crystal structure data for Ca^2+^ bound AnxA2 in solution. This suggests that orientation O5 in which the convex side of the protein faces the membrane represents the most-favored membrane-bound configuration and that Ca^2+^ binding is similar for soluble and membrane bound AnxA2.

Other pairs of orientations (e.g. O5 and O6 or O3 and O9) display similar binding patterns (Fig 4) and, at the same time, have similar binding enthalpies of the AnxA2—membrane interaction (Fig 3). Interestingly, within both pairs the number of relevant Ca^2+^ ions is very different (Fig 3). This suggests that the direct AnxA2 –membrane enthalpy reflects the general binding pattern whereas additional modifications of, e.g., the distance to the membrane via AnxA2—Ca^2+^ interaction are less relevant.

**Fig 6:**
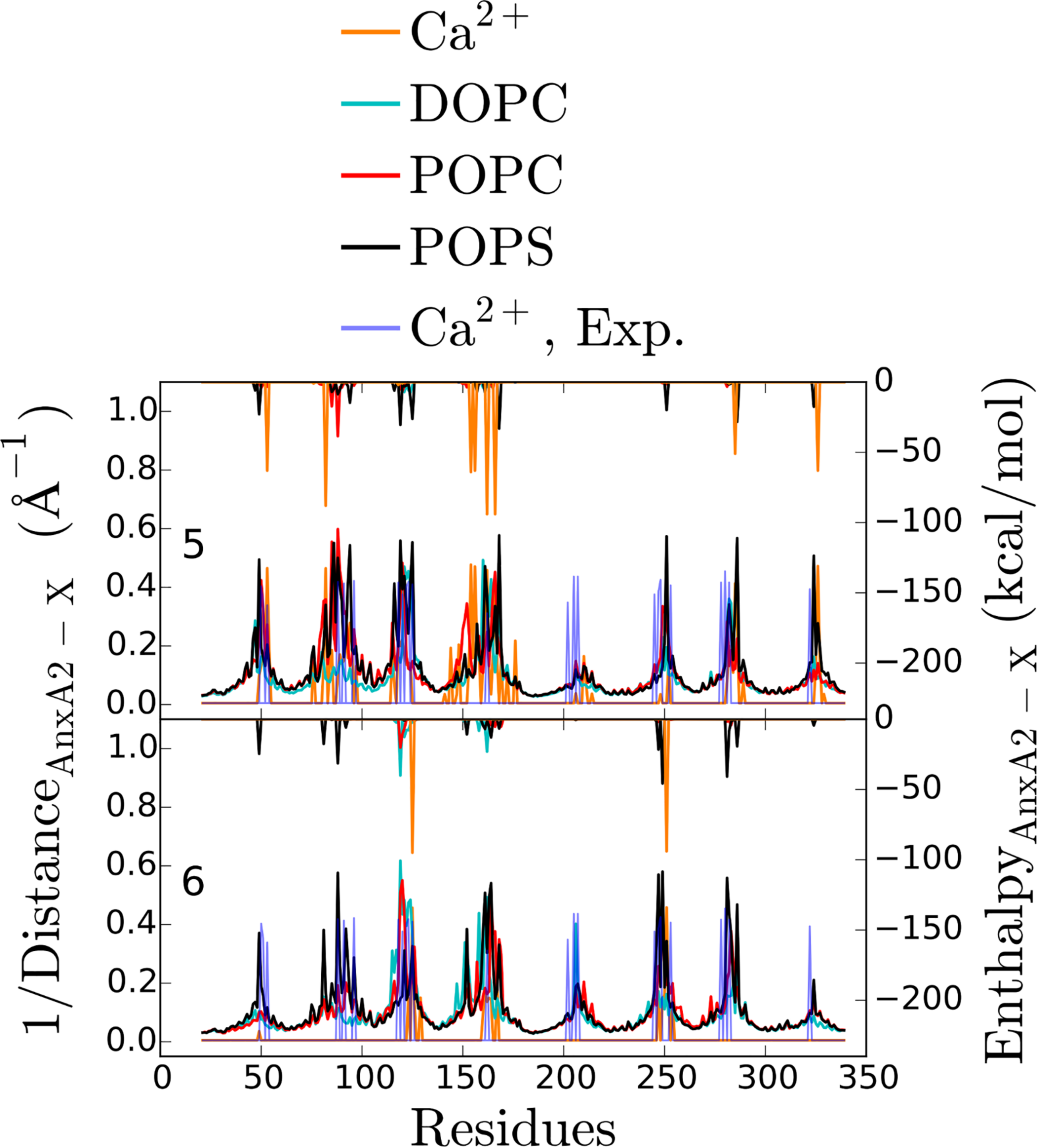
Proximity of AnxA2 residues to lipids and interaction energies for orientations O5 and O6. The inverse of the residue-lipid minimal distances and the residue-Ca^2+^ distances are shown in the bottom curves of each subplot. The upper curves (corresponding to the right axis) show the interaction enthalpies between the same AnxA2 residues and the lipids or Ca^2+^ ions. The data presented are the average values of the last 50 ns of the simulations. The blue peaks are the experimentally determined inverse distances between AnxA2 and Ca^2+^ ions found in the crystal structure of soluble AnxA2.

More details on O5 and O6, including the minimal distances of AnxA2 from certain types of lipids and Ca^2+^ ions as well as the different binding enthalpies, are given in Fig 6. Evidently, AnxA2 predominantly interacts with Ca^2+^ ions and the POPS lipids. For the given concentrations the interaction enthalpies for O5 average to 0, -51, -102, -220 and -550 kcal/mol for CHOL, DOPC, POPC, POPS and Ca^2+^, respectively.

A closer comparison of AnxA2 residues that were bound to Ca^2+^ ions in O5/O6 and those identified as Ca^2+^ coordination sites experimentally [25] reveal some differences. Table 1 provides a list of the Ca^2+^ binding residues for O5/O6 and those seen experimentally:

**Table 1:** Ca^2+^ binding residues in O5/O6 and in experiment [25].

MD simulation	Experiment
O5	Glu53
Glu53	Glu96
Glu82	Glu125
Asp154	Asp162
Glu156	Glu253
Asp162	Glu247
Asp166	Asp322
Asp285	
Asp326	
O6	
Glu125	
Asp251	

The list of binding residues found in O5/O6 is larger than it was reported experimentally. Visual inspection of experimentally identified Ca^2+^ binding sites suggests that some of those sites are unlikely to be preserved with the presence of a membrane. For example 2 Ca^2+^ are bound, on the one hand, to Asp162 and Asp322 residues and, on the other hand, to AB loops of repeats 2 and 4, respectively. However, our observations show that such AB loops gets frequently buried at some extent into the interior of membrane (data not shown) and, therefore, it appears less likely that they could support Ca^2+^ binding in the presence of the membrane as strongly as they do in the membrane free structure.

### AnxA2 binding scenarios

**Fig 7:**
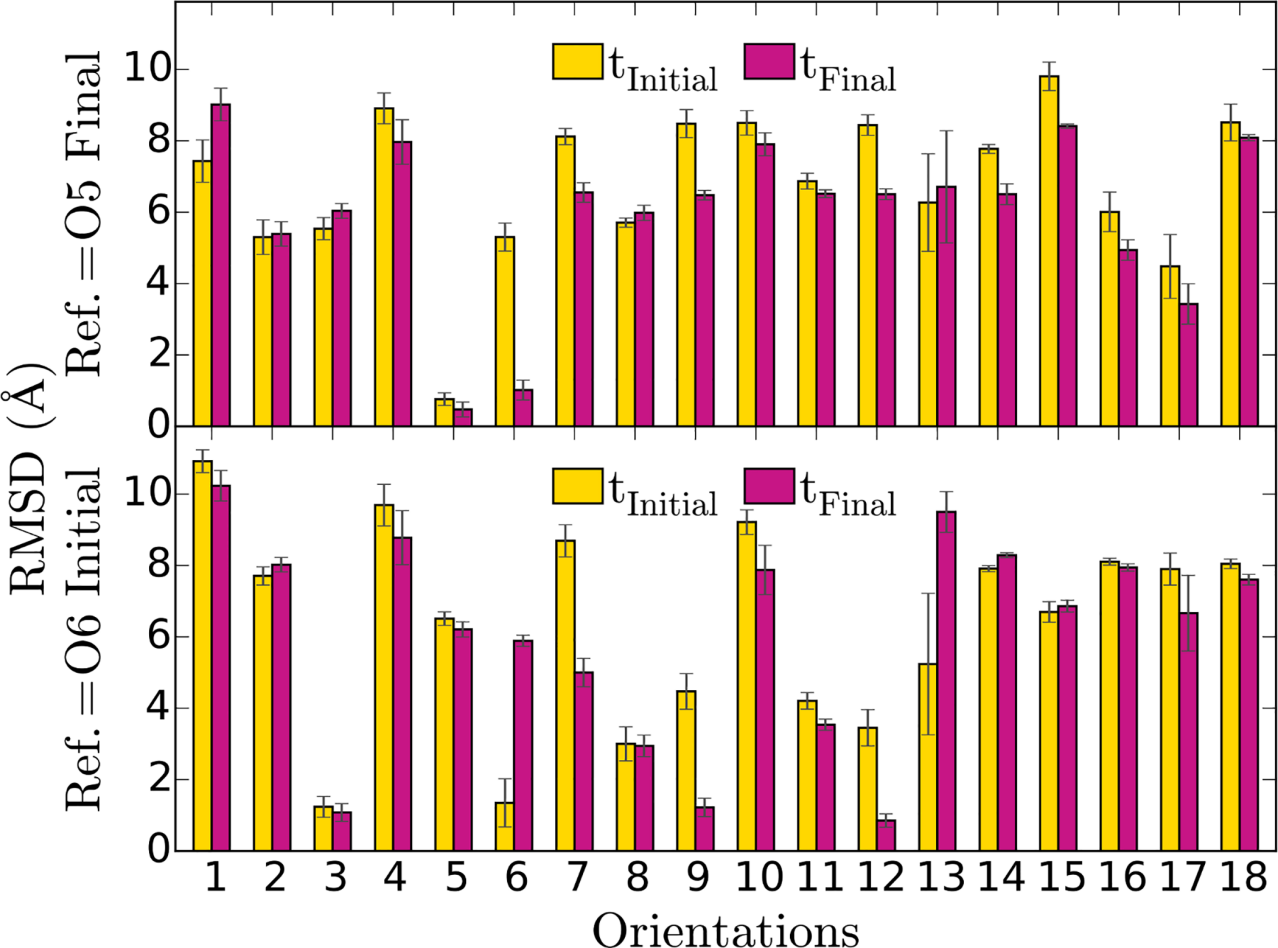
RMSD of AnxA2 coordinates between the 18 orientations and the reference O5 and O6 configurations. In the upper subplot the yellow bars represent the RMSD of the orientations at the initial time averaged over the first 20 ns as compared with the final state of AnxA2 of O5. Correspondingly, the red bars represent the RMSD of the orientations at final time (averaged for the last 20 ns) from the coordinates of the final state of O5. Similarly, the bottom subplot shows the initial and final RMSD relative to the initial configuration of AnxA2 in O6.

To quantify the similarities between the groups shown in Fig 4 we calculated the root mean square differences (RMSD) of the backbone atom coordinates of AnxA2 in the 18 orientations taking as reference coordinates either the final configuration of O5 or the initial configuration of O6. The RMSD effectively accounts for only the coordinate differences of AnxA2 configuration relative to the membrane (i.e. rotational RMSD) as AnxA2 in all the orientations were translated to the center of AnxA2 of the reference structure as well as rotated around the Z axis for the best matching prior to RMSD calculations. The results are shown in Fig 7.

As noted above, the initial O6 orientation approaches the O5 configuration during the 400 ns of simulation time (Fig 7, top). Interestingly, the groups represented by (O3, O9, O12) and (O7, O8, O11) approach the initial configuration of O6 (Fig 7, bottom). This suggests that with longer simulation times the orientations O3, O9, O12, O7, O8 and, likely, O11 and O17 will also resemble O5. In contrast, the members of two other groups, (O1, O4, O10) and (O2, O14, O15, O16, O18) display rather large values of the RMSD with both O5 and O6.

The initial configurations of O14 and O5 differ by a maximum change in the orientation around the x-axis (180° difference). Both configurations show very different properties. Whereas the N-terminal tail in O5 is found at the top and repeats 1 and 2 are facing to the membrane, in O14 the N-terminal tail is located at the bottom of AnxA2 and the repeats 1 and 2 are facing to the direction opposite of the membrane (S1 Fig). More generally, the members of the corresponding group (O2, O14, O15, O16, O18) differ significantly from O5 in terms of the RMSD but also display strong total interaction energies as well as significant values of the AnxA2 –membrane interaction. Binding residues close to the membrane within this group are Glu189, Asp192, Asp230, Asp239, Asp322, Asp338 and Asp339. Bringing together the significant total interaction enthalpies of orientations of this group and the roughly inverted configuration as compared to O5 one may speculate that a dimer of that group with O5 may be a good candidate for the “up-side-down” configuration (see Fig 1).

### AnxA2 and PS interaction

**Fig 8:**
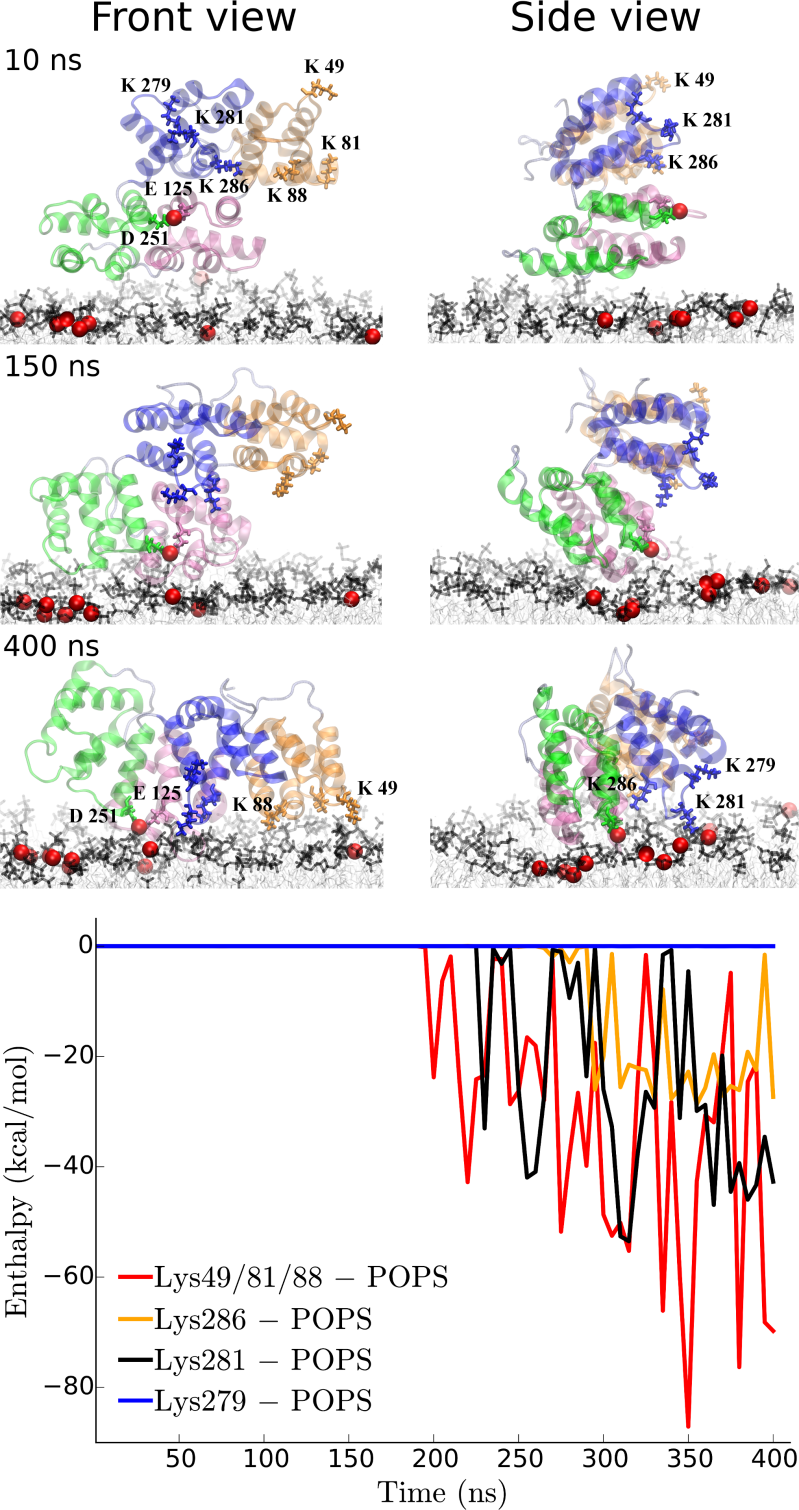
Snapshots of the O6 systems at 50, 150 and 400 ns. The AnxA2 repeats are shown in four different colors as in the previous figures. The Ca^2+^ ions are shown as red spheres, POPS lipids are shown in cyan and other lipids are shown transparent white. The single Ca^2+^ ion bound to AnxA2 at the initial times interacts with residues Glu125 and Asp251 (colored in yellow). Six lysine residues, i.e. Lys49, Lys81, Lys88, Lys279, Lys281 and Lys286, are highlighted in the middle row. The bottom plot shows the temporal total interaction of Lys49/81/88 as well as the interactions of individual Lys279, Lys 281 and Lys286 residues with the POPS lipids.

To address the possibility of a direct interaction between AnxA2 and POPS we focused on configuration O6. The O6 is interesting in two aspects: i) it demonstrates large rotation of AnxA2 toward the favorable configuration O5 under the condition of only a single bound calcium and ii) it serves as a good example of how direct interaction of specific lysine residues with the POPS lipids further changes the O6 configuration towards O5. In Fig 8, snapshots of the initial, intermediate and the final states of membrane-bound AnxA2 are shown for O6. Six lysine residues shown in the second row are of particular interest. At the initial times, AnxA2 interacts with the membrane without the help of Ca^2+^ ions as the only Ca^2+^ bound to Glu125 and Asp251 is positioned too far from the membrane. After about 150 ns the AnxA2-bound Ca^2+^ start to interact with the POPS lipids, giving rise to a rotation of AnxA2 as shown in the side view. At the end of the simulation, AnxA2 appears even more tilted and rotated. At this time, repeat 1 (orange, front view) is pulled toward the membrane because of three lysine residues, Lys49, Lys81 and Lys88 that interact with POPS lipids. Repeat 3 (green) is rotated by almost 90° after 400 ns as compared to the 10 ns configuration (side view). Repeat 4 (blue) is pulled down to the membrane with help of Lys281 and Lys286 and POPS interactions.

It should be noted that the interaction of the six lysine residues with lipids other than POPS or with cholesterol was close to zero for all times (data not shown). The temporal interaction of the lysine residues with lipids can be seen at the bottom of Fig 8. Initiation of the tilt toward the membrane by Lys49/81/88 residues is followed by a rather strong interaction of also Lys281 with POPS lipids (the enthalpy values of the latter is about the half of the sum of the enthalpies of Lys49/81/88).

After 400 ns, residues Lys49/81/88 as well as Lys281 and, partially, Lys286 are in full contact with the POPS lipids. Lys279 and Lys281 were suggested to directly interact with PI(4,5)P_2_ lipids in membrane-bound AnxA2 [28]. Here we observe that Lys279 is not capable to directly interact with POPS lipids. Its preference for PI(4,5)P_2_ is very likely due to the larger head-group of PI(4,5)P_2_, which, however, is not present in the lipid mixture used to simulate the 18 orientations. The observed interaction of Lys281 with POPS suggests that in case of different lipid mixtures, a significant Lys281 –PI(4,5)P_2_ interaction could occur, in agreement with the experimental data.

Except of the discussed six lysine residues there are other lysines that are in direct contact with the membrane (i.e. POPS) in the final O6 configuration. However, presumably, due to either not so favorable localization of POPS or the positioning of the side-chains of those lysine residues their interactions with the membrane were significantly weaker during the simulated period.

### Membranes containing PI(4,5)P_2_

The results shown so far were obtained for membranes containing only POPS as a negatively charged lipid. We additionally simulated AnxA2-membrane-Ca^2+^ systems where different concentrations of PI(4,5)P_2_ lipids were included into membranes. In all of these systems containing PI(4,5)P_2_ AnxA2 was oriented in its favorable O5 configuration with respect to the membrane. Table 2 shows the characteristic concentrations of POPS, PI(4,5)P_2_ and Ca^2+^ of those systems:

**Table 2:** POPS, PI(4,5)P_2_ and Ca^2+^ concentrations in systems A, B and C, respectively, containing PI(4,5)P_2_ lipids.

Systems	POPS (%)	PI(4,5)P_2_ (%)	CA^2+^ (N)
A	21	3	16
B	12	12	16
C	19	5	150

**Fig 9:**
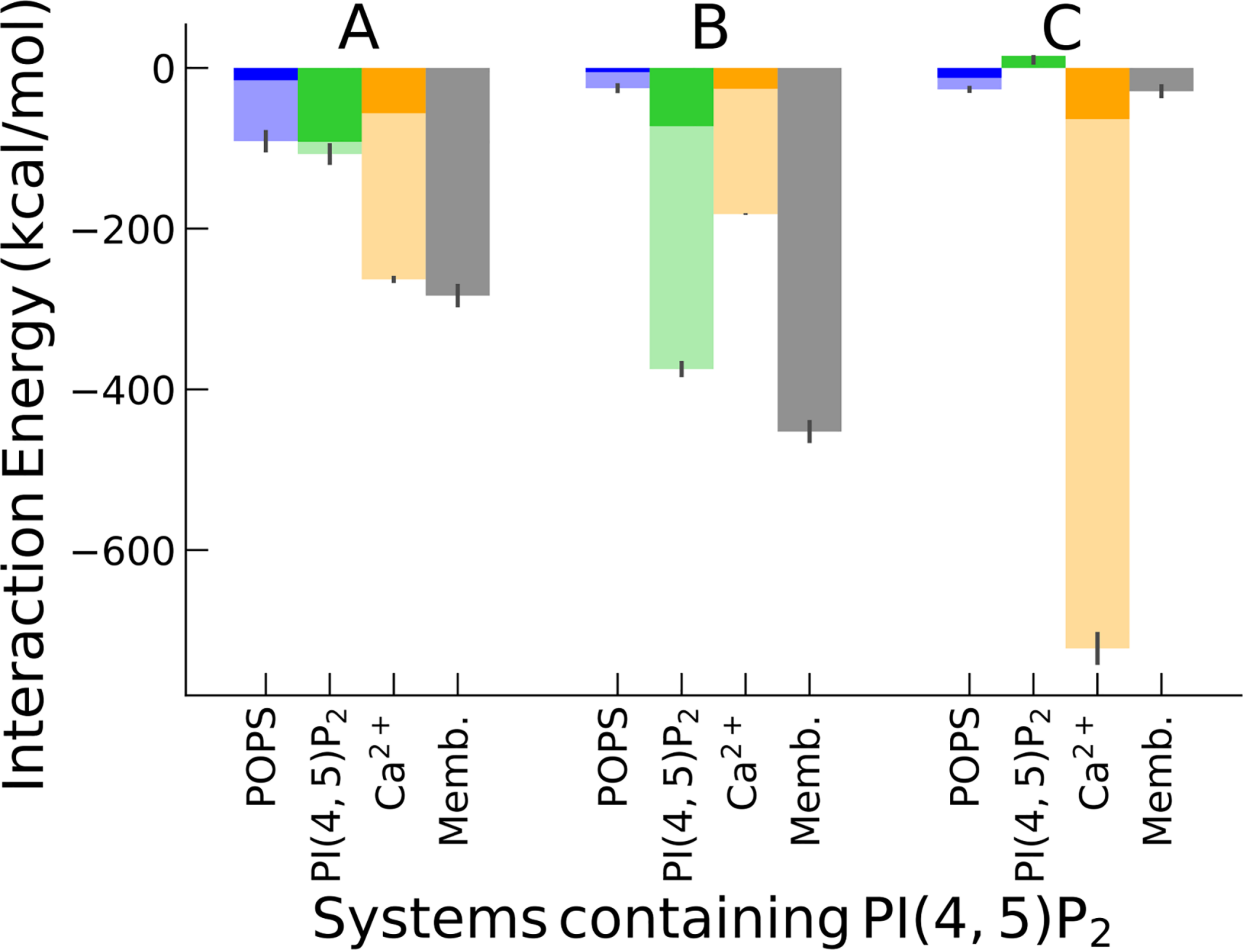
Interaction energies of AnxA2 with POPS, PI(4,5)P_2_, Ca^2+^ and the membrane after 200 ns. The labels A, B and C at the top refer to the systems with different amounts of PI(4,5)P_2_ lipids. The semi-transparent bars display the total interaction energies for POPS, PI(4,5)P_2_ and Ca^2+^ while the bars with solid colors show the interaction energies per given component (i.e. the total interaction energy divided by the number of interacting species).

Due to a large negative charge of PI(4,5)P_2_ (-4) the direct interaction of AnxA2 with the membrane strongly increases. This is particularly relevant in system B where 12% of PI(4,5)P_2_ is present. System C contains a much larger number of Ca^2+^ which corresponds to a 250 μM concentration of Ca^2+^ in experiments (i.e. 1 Ca^2+^ ion per lipid, see S1 Text in Supplementary Material). The interaction energies of AnxA2 with membrane components and Ca^2+^ in those systems are shown in Fig 9.

The 200 ns simulation period is not enough for the systems to reach full equilibration, so that at later times the effects, reported in this work, should be even stronger. Nevertheless, for system A with 3% PI(4,5)P_2_ AnxA2 could interact with only one PI(4,5)P_2_ (six PI(4,5)P_2_ were distributed randomly per bilayer leaflet for this system).while simultaneously interacting with 3–4 Ca^2+^ ions. The total AnxA2-membrane and AnxA2- Ca^2+^ interactions are about the same. On the other hand, in system B the total AnxA2-PI(4,5)P_2_ interaction is significantly stronger while, on the contrary, the AnxA2- Ca^2+^ is weaker than in system A. In case of system C with high concentration of Ca^2+^ the AnxA2-membrane (including PI(4,5)P_2_) interaction is very small. These observations suggest that, at high Ca^2+^ concentrations, the AnxA2-Ca^2+^ interaction dominates over the AnxA2-membrane interaction. In intact cells, however, where the Ca^2+^ concentration is way below 1 μM (similar to system A) the PI(4,5)P_2_ and Ca^2+^ interactions both will support AnxA2-membrane binding.

**Fig 10:**
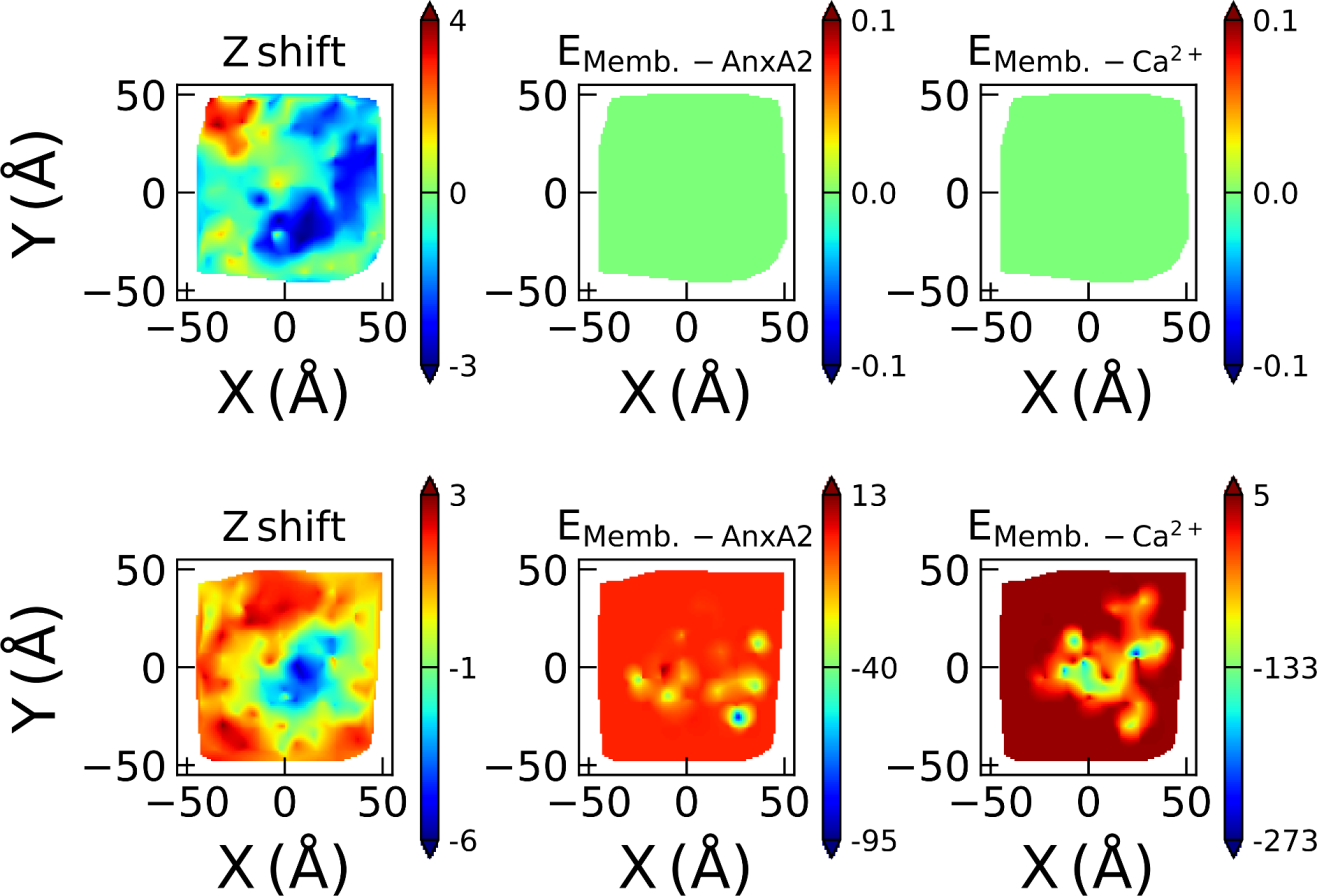
System B: Membrane bending with membrane-AnxA2 and membrane-Ca^2+^ interactions. The upper and lower rows represent the membrane leaflets which are non-interacting and interacting with AnxA2, respectively. The first column represents the difference in the Z direction of the phosphor P atoms in all lipids relative to the average Z value in Å. The second column displays the total interaction energy of membrane-AnxA2 pairs while the last column represents the total interaction energy of the membrane-Ca^2+^ pairs. The energies in the second and third columns are in units kcal/mol. The points in the X and Y planes reflect the coordinates of the P atoms of the lipids.

In system B the AnxA2-membrane interaction through PI(4,5)P_2_ lipids is the strongest but can still be compared with the membrane-Ca^2+^ interactions. Fig 10 shows the membrane curvature as reflected by the z coordinates of the lipids’ phosphor atoms P relative to the average z value of all P atoms in the corresponding leaflet as well as the interaction energies of membrane-AnxA2 and membrane-Ca^2+^ pairs, respectively.

Obviously, the bending of the leaflet represented in the upper row is a result of the bending of the leaflet in the bottom row where the membrane and most of Ca^2+^ are localized. Interestingly, the membrane regions with negative curvature correlate with membrane-Ca^2+^ interactions much better than with membrane-AnxA2 interactions. Similar behaviors can be observed for systems A and C shown in S2 Fig. Table 3 shows the correlation coefficients of curvature and the interaction of lipids with either AnxA2 or Ca^2+^. It can be noted that for systems A and B the correlation coefficient of the membrane leaflet with AnxA2 is 0.4–0.5 for membrane-Ca^2+^ interaction while the influence of membrane-AnxA2 on the curvature is close to zero. In most of in-vitro experiments as well as in MD simulations a high Ca^2+^ concentration was used. As a result, the large number of Ca^2+^ ions mask the direct membrane-AnxA2 interaction. Our results suggest that the membrane bending occurs due to membrane-Ca^2+^ interactions and is not driven by the interactions with the convex face of AnxA2.

**Table 3:** Correlation coefficients of membrane bending and interaction energies in systems A, B and C.

Leaflets / Pairs	Correlation coefficients Systems		
	A	B	C
AnxA2-non-interacting / Z shift vs. E_Memb.-AnxA2_	N/A	N/A	N/A
AnxA2-non-interacting / Z shift vs. E_Memb.-calcium_	NA	N/A	-0.18
AnxA2-interacting / Z shift vs. E_Memb.-AnxA2_	-0.03	0.08	-0.21
AnxA2-interacting / Z shift vs. E_Memb.-calcium_	0.41	0.52	-0.01

**Fig 11:**
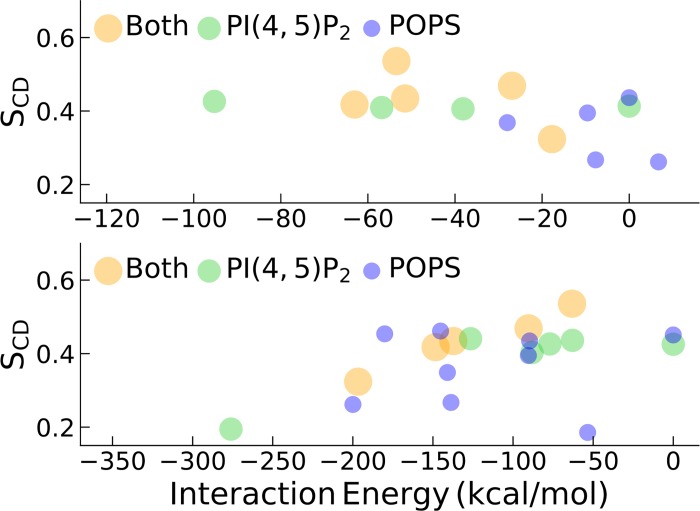
System B: Lipid order parameters S_CD_ as a function of membrane-AnxA2 and membrane-Ca^2+^ interactions. In the upper and lower plots the horizontal axes represent membrane-AnxA2 and membrane-Ca^2+^ interaction energies, respectively. Shown are the average values for the last 20 ns of the 200 ns simulation. The PI(4,5)P_2_ and POPS lipids which significantly interacted with both AnxA2 as well as with Ca^2+^ ions are shown in yellow.

We also analyzed the correlation of the order parameter (S_CD_) of membrane lipids with the interaction strength with either AnxA2 or Ca^2+^ ions. Fig 11 shows the dependence of the average order parameter on the respective interaction energy for system B. Those lipids which significantly interacted with both AnxA2 and Ca^2+^ ions are shown separately with yellow circles. Such lipids were predominantly PI(4,5)P_2_ lipids.

Interestingly, a stronger interaction of the membrane with AnxA2 gives rise to an increase of the order parameter of the corresponding interacting lipids while, on the contrary, a stronger interaction with Ca^2+^ results in the opposite effect. Somewhat weaker but similar effects can be observed for system A in S3 Fig. For system C the bilayer seems to be saturated by Ca^2+^ (membrane-AnxA2 interaction is negligible) and the order parameter does not show a particular dependence on the interaction with Ca^2+^ (S3 Fig). In recent work Chen and co-workers have found that the lipid order was decreased upon interaction with annexin A5 [38]. This apparent discrepancy may be a result of structural differences between AnxA2 and annexin A5. Another reason can be the fact that Chen et al. used a high Ca^2+^ concentration which played a primary role in annexin A5 and membrane interaction even when annexin A5 was positioned very close to the membrane. Thus, the interaction with the annexin A5 could be dominated by the presence of Ca^2+^. This would be fully compatible with the present results where we could individually analyze both contributions.

Two additional results are helpful for our systematic analysis of the membrane-AnxA2 interaction. (1) When simulating AnxA2 and 12 Ca^2+^ ions together with a neutral membrane i.e. with only DOPC and POPC lipids, no binding of AnxA2 was observed. The protein was detached from the membrane surface together with most of the calcium ions (data not shown). Thus, charged lipids are indeed essential for the membrane-AnxA2 binding. (2) When placing the full length AnxA2 with reconstructed first 20 residues of its N-terminal tail toward the membrane (with composition DOPC^19%^:POPC^38%^:POPS^19%^:PI(4,5)P_2_^5%^:CHOL^19%^) we also observed fast detachment within the first 50 ns (data not shown). Thus, a potential interaction of the membrane with the N-terminal tail of AnxA2 is by far too weak. This agrees with the suggestion that AnxA2 core is solely responsible for membrane binding and lipid segregation [28].

## Discussion

Our MD simulations of different AnxA2 orientations on a membrane consisting of DOPC, POPC, POPS and cholesterol have yielded for the first time atomistic information concerning the structure, enthalpy, orientation, and lipid headgroup binding characterizing the AnxA2—membrane interaction. Several open questions in the field can now be addressed in light of our observations. First, crystal structures of AnxA2 and other annexins in solution have revealed that the proteins fold into compact discs with one side, the convex side, harboring annexin-type Ca^2+^ binding sites. It has been speculated but never shown that this side faces the membrane in membrane-bound annexins. Our simulations indicate that this is indeed the case as orientation O5 resembling the configuration with annexin-type Ca^2+^ binding sites facing the membrane is the most favorable orientation of the protein on membranes in terms of the enthalpic interaction.

Second, while the formation of AnxA2 dimers in an „up-side-down”configuration has been discussed but never proven [25], we now present evidence that such dimeric interaction between two AnxA2 monomers in the „up-side-down”configuration could exist when Ca^2+^ ions are present. Our simulations end in two main AnxA2 –membrane configurations. The first one is the most favorable configuration represented by O5 and the closely resembling O6 orientation (also covering orientation groups such as O8 and O9) and the second is represented by O14 and its group that together with O5 could form an „up-side-down”dimer. Of course, the present simulations cannot elucidate the properties of the direct AnxA2-AnxA2-interaction in the “up-side-down” configuration.

Third, we demonstrate that AnxA2 can interact with the POPS lipids present in our mixture without the need to involve Ca^2+^ ions. This direct interaction was observed in the induced rotation of AnxA2 from the O6 to the O5 configuration in a Ca^2+^ depleted environment (only a single AnxA2-Ca^2+^ pair). Such Ca^2+^ independent interactions were not observed in many experimental lipid binding studies [28, 46], but possible AnxA2-membrane interactions that could occur in the absence of Ca^2+^-ions had been discussed before [47]. This discrepancy is, possibly, related to the rather unstable interaction between AnxA2 and POPS lipids due to diffusion effects of POPS lipids and the high mobility of the lysine side-chains which render the experimental observation of binding effects more difficult.

Fourth, we emphasize the role of Glu125/Asp251 as a binding site for Ca^2+^ at low Ca^2+^ concentration. Binding of Glu125 and Asp251 to a single Ca^2+^ ion on one hand and binding of that Ca^2+^ to POPS or PI(4,5)P_2_ lipids on the other hand strongly stabilizes AnxA2 in the favorable O5 configuration (Fig 8) and shows significant interactions in the O6 configuration (Fig 3). In contrast to Glu125/Asp251 the other Ca^2+^ binding sites revealed in O5 are localized more on periphery of the molecule which seems to require several Ca^2+^ to be bound at the same time for effective membrane interaction in O5 configuration (see S1 Fig, configuration O2 where only one of the binding sites were interacting with Ca^2+^ which resulted in tilting of AnxA2).

Fifth, the negative curvature of the membrane, which is observed in the AnxA2/ Ca^2+^/membrane simulations, seems to be primarily related to the membrane-Ca^2+^ interaction rather than the convex membrane-binding surface of AnxA2. Clearly, once AnxA2 binds to Ca^2+^ ions, the convex face of the former plays a positive and likely important role in membrane bending. However, AnxA2 seems to serve more as a platform for fixation of the negative curvature of the membrane induced by Ca^2+^ ions. This agrees with a recent study of influence of Ca^2+^ on negatively charged bilayers containing POPS and PI(4,5)P_2_ lipids [19].

Sixth, the direct interaction of AnxA2 with the membrane induces an ordering of interacting lipids while the membrane-Ca^2+^ interaction distorts the lipid order. This was observed in our MD systems only at low Ca^2+^ concentrations. At high Ca^2+^ concentrations the direct AnxA2-membrane interaction is minimal as it is almost completely masked by much stronger membrane-Ca^2+^ interactions. To the best of our knowledge, the AnxA2-induced ordering effect of lipids is reported here for the first time, possibly, because most of the previous annexin studies used high Ca^2+^ concentration.

In more general terms, the AnxA2 –membrane binding is an interesting example where different cooperative effects are important. First, as seen for the time evolution of the initial O6 configuration, the simultaneous rotation and the clustering of the negatively charged lipids gives rise to an increased stability, rendering the O6 configuration more O5-type. Second, the presence of negatively charged lipids will increase the attraction of Ca^2+^ ending up in the stable O5 configuration, including the large number of calcium ions. Molecular Dynamics simulations can help to elucidate the details of these mechanisms.

## Materials and methods

### Initial structure preparation

The system consisting of a membrane of the composition POPC:DOPC:POPS:CHOL at relative concentrations of 0.38:0.19:0.24:0.19 (152 POPC, 76 DOPC, 96 POPS and 76 CHOL molecules i.e. 200 molecules per leaflet) and of monomeric AnxA2 (PDB code 1W7B) [25] was prepared using the CHARMM-GUI Web-based graphical interface [48, 49] and CHARMM36 force field [50, 51]. This structure generated by CHARMM-GUI is further manipulated with the CHARMM package [52]. First the water molecules were removed from the unit cell and the AnxA2 was centered on the membrane surface such that its first and second principle axes were parallel to X and Y directions, respectively, i.e. AnxA2 was lying in a very flat position over the membrane. Then AnxA2 was moved away in Z direction to a large enough distance (100 nm). The resulting initial structure served as a basis for generation of all the relative orientations of AnxA2 in respect to membrane.

### Generation of the 18 AnxA2 orientations

To generate the different initial orientations of AnxA2 in a systematic way, the initial structure of AnxA2 was rotated around its center of mass, first around the X axis and subsequently around the Y axis. After rotation of AnxA2 in each of the 18 orientations AnxA2 was dragged in Z direction back to the membrane such that the minimal distance between the membrane and AnxA2 became about 6 Å. The respective angles of rotations are shown in Table 4.

**Table 4:** 18 orientations of AnxA2 generated by rotation of the initial structure around X or Y axes.

Rotation angle around X axis	Rotation angle around Y axis				
	-60°		0°	60°	
**0°**	O1		O2	O3	
**60°**	O4		O5	O6	
**120°**	O7		O8	O9	
**180°**	O10		O11	O12	
**240°**	O13		O14	O15	
**300°**	O16		O17	O18	

All the 18 systems were solvated with the TIP3P type water molecules. 12 water molecules were randomly selected within the region between membrane and AnxA2 and replaced by Ca^2+^ ions. The final system sizes of each 18 systems on average was 135 x 135 x 150 Å. With the presence of negatively charged lipids such as POPS the membrane-Ca^2+^ binding takes place at sub-nanosecond times. Because of this fast kinetics, the AnxA2-membrane interaction studied in our MD simulations can be considered as starting at a condition where Ca^2+^ is bound to the membrane. To finalize the preparations of the 18 systems the required amount of chloride and sodium ions were added to each system to obtain ~150 mM salt concentration and to guarantee neutral charge. Each of the 18 systems on average contained 190000 atoms.

Additionally, three systems A, B and C were prepared with PI(4,5)P_2_ lipids added to membranes. The first two systems A and B contained 3% and 12% of PI(4,5)P_2_ lipids (84 POPS and 12 PI(4,5)P_2_ lipids for system A and 48 POPS and 48 PI(4,5)P_2_ for system B) and 16 Ca^2+^ ions (i.e. the concentrations of POPS lipids in these systems were decreased to 21% and 12%, respectively). The third system C with 5% PI(4,5)P_2_ and 19% POPS (76 POPS and 20 PI(4,5)P_2_ lipids) included much higher Ca^2+^ concentrations with a total number of 150 Ca^2+^ ions. In all system A, B and C AnxA2 was placed above the membrane at the predefined orientation O5 and at a 2 Å minimal distance from the membrane. Here the minimal distance from the membrane was chosen smaller to account for the larger head groups of PI(4,5)P_2_ lipids to keep the direct interaction of AnxA2 with the membrane strong enough.

### Simulation settings

The MD simulations were performed with the Gromacs package [53–55] and analyzed by the CHARMM program. The electrostatic and van der Waals interactions were calculated through the particle mesh Ewald [56] and cutoff schemes, respectively, with cutoff distance of 1.2 nm. The NPT ensemble, semiisotropic pressure tensor of 1 bar, Parrinello-Rahman pressure coupling [57] and Nosé–Hoover temperature coupling [58, 59] methods were used together with the time step of 2 fs. Each of the 18 systems were simulated at 293 K temperature for 400 ns. The three systems A, B and C with PI(4,5)P_2_ were simulated at 310 K for 200 ns.

## Supporting information

S1 Fig The initial and final configurations of 18 systems. Each pair shows the initial and final configurations of AnxA2 in respective system. The four repeats of AnxA2 are shown in different colors: repeats 1 (Ala35 –Lys104), 2 (Ala107 –Lys176), 3 (Asp187 –Ile263) and 4 (Lys266 –Cys335). The experimentally found Ca^2+^ binding Asp or Glu residues are shown in black. The Ca^2+^ ions are shown as red spheres.
(PDF)

S2 Fig Membrane bending with membrane-AnxA2 and membrane-Ca^2+^ interactions. (A) System A. (B) System C. The upper and lower rows in each subplot represent membrane leaflets which are non-interacting and interacting with AnxA2, respectively. The first column represents the difference in the Z direction of the phosphor P atoms in all lipids relative to the average Z value in angstroms. The second column displays the total interaction energy of membrane-AnxA2 pair while the last column represent the total interaction energy of the membrane-Ca^2+^ pair. The energies in the second and third columns are in units of kcal/mol. The points in the X and Y planes reflect the coordinates of the P atoms of the lipids.
(PDF)

S3 Fig Lipid order parameters S_CD_ as a function of membrane-AnxA2 and membrane-Ca^2+^ interactions. (A) System A. (B) System C. In the upper and lower plots the horizontal axes represent membrane-AnxA2 and membrane-Ca^2+^ interaction energies, respectively. Shown are the average values for the last 20 ns of the 200 ns simulation. The PI(4,5)P_2_ and POPS lipids which significantly interacted with both AnxA2 as well as with Ca^2+^ ions are shown in yellow.
(PDF)

S1 Text Concentration of Ca^2+^ for system C. (PDF)

## References

[1] Gerke V, Moss SE. Annexins: from structure to function. Physiol Rev. 2002;82(2):331–71. doi: 10.1152/physrev.00030.2001 11917092 10.1152/physrev.00030.2001

[2] Raynal P, Pollard HB. Annexins: the problem of assessing the biological role for a gene family of multifunctional calcium- and phospholipid-binding proteins. Biochim Biophys Acta. 1994;1197(1):63–93. 8155692 10.1016/0304-4157(94)90019-1

[3] Gerke V, Creutz CE, Moss SE. Annexins: linking Ca2+ signalling to membrane dynamics. Nat Rev Mol Cell Biol. 2005;6(6):449–61. doi: 10.1038/nrm1661 15928709 10.1038/nrm1661

[4] Koerdt SN, Gerke V. Annexin A2 is involved in Ca2+-dependent plasma membrane repair in primary human endothelial cells. Biochim Biophys Acta. 2017;1864(6):1046–53. doi: 10.1016/j.bbamcr.2016.12.007 27956131 10.1016/j.bbamcr.2016.12.007

[5] Gerke V. Annexins A2 and A8 in endothelial cell exocytosis and the control of vascular homeostasis. Biol Chem. 2016;397(10):995–1003. doi: 10.1515/hsz-2016-0207 27451994 10.1515/hsz-2016-0207

[6] Gerke V, Weber K. Calcium-dependent conformational changes in the 36-kDa subunit of intestinal protein I related to the cellular 36-kDa target of Rous sarcoma virus tyrosine kinase. J Biol Chem. 1985;260(3):1688–95. 2981869

[7] Thiel C, Osborn M, Gerke V. The tight association of the tyrosine kinase substrate annexin II with the submembranous cytoskeleton depends on intact p11- and Ca(2+)-binding sites. J Cell Sci. 1992;103 (Pt 3):733–42. 1478969 10.1242/jcs.103.3.733

[8] Zokas L, Glenney JR Jr. The calpactin light chain is tightly linked to the cytoskeletal form of calpactin I: studies using monoclonal antibodies to calpactin subunits. J Cell Biol. 1987;105(5):2111–21. 2960683 10.1083/jcb.105.5.2111 PMC2114835

[9] Waisman DM. Annexin II tetramer: structure and function. Mol Cell Biochem. 1995;149–150:301–22. 8569746 10.1007/BF01076592

[10] Chasserot-Golaz S, Vitale N, Umbrecht-Jenck E, Knight D, Gerke V, Bader MF. Annexin 2 promotes the formation of lipid microdomains required for calcium-regulated exocytosis of dense-core vesicles. Mol Biol Cell. 2005;16(3):1108–19. doi: 10.1091/mbc.E04-07-0627 15635098 10.1091/mbc.E04-07-0627 PMC551477

[11] Rety S, Sopkova J, Renouard M, Osterloh D, Gerke V, Tabaries S, et al The crystal structure of a complex of p11 with the annexin II N-terminal peptide. Nat Struct Biol. 1999;6(1):89–95. doi: 10.1038/4965 9886297 10.1038/4965

[12] Ayala-Sanmartin J, Zibouche M, Illien F, Vincent M, Gallay J. Insight into the location and dynamics of the annexin A2 N-terminal domain during Ca(2+)-induced membrane bridging. Biochim Biophys Acta. 2008;1778(2):472–82. doi: 10.1016/j.bbamem.2007.11.004 18068113 10.1016/j.bbamem.2007.11.004

[13] Johnsson N, Vandekerckhove J, Van Damme J, Weber K. Binding sites for calcium, lipid and p11 on p36, the substrate of retroviral tyrosine-specific protein kinases. FEBS Lett. 1986;198(2):361–4. 2937654 10.1016/0014-5793(86)80437-9

[14] Glenney J. Phospholipid-dependent Ca2+ binding by the 36-kDa tyrosine kinase substrate (calpactin) and its 33-kDa core. J Biol Chem. 1986;261(16):7247–52. 2940239

[15] Jost M, Weber K, Gerke V. Annexin II contains two types of Ca(2+)-binding sites. Biochem J. 1994;298 Pt 3:553–9. 7908188 10.1042/bj2980553 PMC1137894

[16] Melcrova A, Pokorna S, Pullanchery S, Kohagen M, Jurkiewicz P, Hof M, et al The complex nature of calcium cation interactions with phospholipid bilayers. Sci Rep. 2016;6:38035 doi: 10.1038/srep38035 27905555 10.1038/srep38035 PMC5131315

[17] Magarkar A, Jurkiewicz P, Allolio C, Hof M, Jungwirth P. Increased Binding of Calcium Ions at Positively Curved Phospholipid Membranes. J Phys Chem Lett. 2017;8(2):518–23. doi: 10.1021/acs.jpclett.6b02818 28067523 10.1021/acs.jpclett.6b02818

[18] Kucerka N, Dushanov E, Kholmurodov KT, Katsaras J, Uhrikova D. Calcium and Zinc Differentially Affect the Structure of Lipid Membranes. Langmuir: the ACS journal of surfaces and colloids. 2017;33(12):3134–41. doi: 10.1021/acs.langmuir.6b03228 28277666 10.1021/acs.langmuir.6b03228

[19] Graber ZT, Shi Z, Baumgart T. Cations induce shape remodeling of negatively charged phospholipid membranes. Phys Chem Chem Phys. 2017;19(23):15285–95. doi: 10.1039/c7cp00718c 28569910 10.1039/c7cp00718c PMC5562360

[20] Rescher U, Ruhe D, Ludwig C, Zobiack N, Gerke V. Annexin 2 is a phosphatidylinositol (4,5)-bisphosphate binding protein recruited to actin assembly sites at cellular membranes. J Cell Sci. 2004;117(Pt 16):3473–80. doi: 10.1242/jcs.01208 15226372 10.1242/jcs.01208

[21] Menke M, Gerke V, Steinem C. Phosphatidylserine membrane domain clustering induced by annexin A2/S100A10 heterotetramer. Biochemistry (Mosc). 2005;44(46):15296–303. doi: 10.1021/bi051585i 16285733 10.1021/bi051585i

[22] Glenney JR Jr., Tack B, Powell MA. Calpactins: two distinct Ca++-regulated phospholipid- and actin-binding proteins isolated from lung and placenta. J Cell Biol. 1987;104(3):503–11. 2950118 10.1083/jcb.104.3.503 PMC2114563

[23] Blackwood RA, Ernst JD. Characterization of Ca2(+)-dependent phospholipid binding, vesicle aggregation and membrane fusion by annexins. Biochem J. 1990;266(1):195–200. 2138016 10.1042/bj2660195 PMC1131114

[24] Ayala-Sanmartin J, Vincent M, Sopkova J, Gallay J. Modulation by Ca(2+) and by membrane binding of the dynamics of domain III of annexin 2 (p36) and the annexin 2-p11 complex (p90): implications for their biochemical properties. Biochemistry (Mosc). 2000;39(49):15179–89. 11106497 10.1021/bi000501x

[25] Rosengarth A, Luecke H. Annexin A2. Does it induce membrane aggregation by a new multimeric state of the protein? Annexins. 2004;1(2):129–36.

[26] Lambert O, Gerke V, Bader MF, Porte F, Brisson A. Structural analysis of junctions formed between lipid membranes and several annexins by cryo-electron microscopy. J Mol Biol. 1997;272(1):42–55. doi: 10.1006/jmbi.1997.1183 9299336 10.1006/jmbi.1997.1183

[27] Lambert O, Cavusoglu N, Gallay J, Vincent M, Rigaud JL, Henry JP, et al Novel organization and properties of annexin 2-membrane complexes. J Biol Chem. 2004;279(12):10872–82. doi: 10.1074/jbc.M313657200 14701819 10.1074/jbc.M313657200

[28] Drucker P, Pejic M, Galla HJ, Gerke V. Lipid segregation and membrane budding induced by the peripheral membrane binding protein annexin A2. J Biol Chem. 2013;288(34):24764–76. doi: 10.1074/jbc.M113.474023 23861394 10.1074/jbc.M113.474023 PMC3750172

[29] Pigault C, Follenius-Wund A, Lux B, Gerard D. A fluorescence spectroscopy study of the calpactin I complex and its subunits p11 and p36: calcium-dependent conformation changes. Biochim Biophys Acta. 1990;1037(1):106–14. 2136798 10.1016/0167-4838(90)90108-r

[30] Meers P. Location of tryptophans in membrane-bound annexins. Biochemistry (Mosc). 1990;29(13):3325–30. 2139793 10.1021/bi00465a025

[31] Concha NO, Head JF, Kaetzel MA, Dedman JR, Seaton BA. Rat annexin V crystal structure: Ca(2+)-induced conformational changes. Science. 1993;261(5126):1321–4. 8362244 10.1126/science.8362244

[32] Sopkova J, Gallay J, Vincent M, Pancoska P, Lewit-Bentley A. The dynamic behavior of annexin V as a function of calcium ion binding: a circular dichroism, UV absorption, and steady-state and time-resolved fluorescence study. Biochemistry (Mosc). 1994;33(15):4490–9. 8161503 10.1021/bi00181a008

[33] Sopkova J, Vincent M, Takahashi M, Lewit-Bentley A, Gallay J. Conformational flexibility of domain III of annexin V studied by fluorescence of tryptophan 187 and circular dichroism: the effect of pH. Biochemistry (Mosc). 1998;37(34):11962–70. doi: 10.1021/bi980773o 9718321 10.1021/bi980773o

[34] Shesham RD, Bartolotti LJ, Li Y. Molecular dynamics simulation studies on Ca2+ -induced conformational changes of annexin I. Protein Eng Des Sel. 2008;21(2):115–20. doi: 10.1093/protein/gzm094 18283055 10.1093/protein/gzm094

[35] Donohue MP, Bartolotti LJ, Li Y. The N-terminal of annexin A1 as a secondary membrane binding site: a molecular dynamics study. Proteins. 2014;82(11):2936–42. doi: 10.1002/prot.24623 24913225 10.1002/prot.24623

[36] Simpkins B, Donohue MP, Li Y. Molecular dynamic studies on the impact of mutations on the structure, stability, and N-terminal orientation of annexin A1: implications for membrane aggregation. Proteins. 2014;82(12):3327–34. doi: 10.1002/prot.24684 25204809 10.1002/prot.24684

[37] Merzel F, Hodoscek M, Janezic D, Sanson A. New force field for calcium binding sites in annexin-membrane complexes. J Comput Chem. 2006;27(4):446–52. doi: 10.1002/jcc.20340 16419147 10.1002/jcc.20340

[38] Chen Z, Mao Y, Yang J, Zhang T, Zhao L, Yu K, et al Characterizing the binding of annexin V to a lipid bilayer using molecular dynamics simulations. Proteins. 2014;82(2):312–22. doi: 10.1002/prot.24389 23934928 10.1002/prot.24389

[39] Miyagi A, Chipot C, Rangl M, Scheuring S. High-speed atomic force microscopy shows that annexin V stabilizes membranes on the second timescale. Nat Nanotechnol. 2016;11(9):783–90. doi: 10.1038/nnano.2016.89 27271964 10.1038/nnano.2016.89

[40] Almeida PF, Best A, Hinderliter A. Monte Carlo simulation of protein-induced lipid demixing in a membrane with interactions derived from experiment. Biophys J. 2011;101(8):1930–7. doi: 10.1016/j.bpj.2011.09.015 22004747 10.1016/j.bpj.2011.09.015 PMC3192976

[41] Fisette O, Paslack C, Barnes R, Isas JM, Langen R, Heyden M, et al Hydration Dynamics of a Peripheral Membrane Protein. J Am Chem Soc. 2016;138(36):11526–35. doi: 10.1021/jacs.6b07005 27548572 10.1021/jacs.6b07005 PMC5519773

[42] Comeau SR, Gatchell DW, Vajda S, Camacho CJ. ClusPro: a fully automated algorithm for protein-protein docking. Nucleic Acids Res. 2004;32(Web Server issue):W96–9. doi: 10.1093/nar/gkh354 15215358 10.1093/nar/gkh354 PMC441492

[43] Pierce BG, Wiehe K, Hwang H, Kim BH, Vreven T, Weng Z. ZDOCK server: interactive docking prediction of protein-protein complexes and symmetric multimers. Bioinformatics. 2014;30(12):1771–3. doi: 10.1093/bioinformatics/btu097 24532726 10.1093/bioinformatics/btu097 PMC4058926

[44] Roberts VA, Thompson EE, Pique ME, Perez MS, Ten Eyck LF. DOT2: Macromolecular docking with improved biophysical models. J Comput Chem. 2013;34(20):1743–58. doi: 10.1002/jcc.23304 23695987 10.1002/jcc.23304 PMC4370774

[45] Singharoy A, Barragan AM, Thangapandian S, Tajkhorshid E, Schulten K. Binding Site Recognition and Docking Dynamics of a Single Electron Transport Protein: Cytochrome c2. J Am Chem Soc. 2016;138(37):12077–89. doi: 10.1021/jacs.6b01193 27508459 10.1021/jacs.6b01193 PMC5518707

[46] Swairjo MA, Concha NO, Kaetzel MA, Dedman JR, Seaton BA. Ca(2+)-bridging mechanism and phospholipid head group recognition in the membrane-binding protein annexin V. Nat Struct Biol. 1995;2(11):968–74. 7583670 10.1038/nsb1195-968

[47] Jost M, Zeuschner D, Seemann J, Weber K, Gerke V. Identification and characterization of a novel type of annexin-membrane interaction: Ca2+ is not required for the association of annexin II with early endosomes. J Cell Sci. 1997;110 (Pt 2):221–8. 9044052 10.1242/jcs.110.2.221

[48] Jo S, Kim T, Iyer VG, Im W. CHARMM-GUI: a web-based graphical user interface for CHARMM. J Comput Chem. 2008;29(11):1859–65. doi: 10.1002/jcc.20945 18351591 10.1002/jcc.20945

[49] Wu EL, Cheng X, Jo S, Rui H, Song KC, Davila-Contreras EM, et al CHARMM-GUI Membrane Builder toward realistic biological membrane simulations. J Comput Chem. 2014;35(27):1997–2004. doi: 10.1002/jcc.23702 25130509 10.1002/jcc.23702 PMC4165794

[50] Klauda JB, Venable RM, Freites JA, O'Connor JW, Tobias DJ, Mondragon-Ramirez C, et al Update of the CHARMM all-atom additive force field for lipids: validation on six lipid types. J Phys Chem B. 2010;114(23):7830–43. doi: 10.1021/jp101759q 20496934 10.1021/jp101759q PMC2922408

[51] Huang J, MacKerell AD Jr. CHARMM36 all-atom additive protein force field: validation based on comparison to NMR data. J Comput Chem. 2013;34(25):2135–45. doi: 10.1002/jcc.23354 23832629 10.1002/jcc.23354 PMC3800559

[52] Brooks BR, Brooks CL 3rd, Mackerell AD Jr., Nilsson L, Petrella RJ, Roux B, et al CHARMM: the biomolecular simulation program. J Comput Chem. 2009;30(10):1545–614. doi: 10.1002/jcc.21287 19444816 10.1002/jcc.21287 PMC2810661

[53] Van Der Spoel D, Lindahl E, Hess B, Groenhof G, Mark AE, Berendsen HJ. GROMACS: fast, flexible, and free. J Comput Chem. 2005;26(16):1701–18. doi: 10.1002/jcc.20291 16211538 10.1002/jcc.20291

[54] Hess B, Kutzner C, van der Spoel D, Lindahl E. GROMACS 4: Algorithms for Highly Efficient, Load-Balanced, and Scalable Molecular Simulation. J Chem Theory Comput. 2008;4(3):435–47. doi: 10.1021/ct700301q 26620784 10.1021/ct700301q

[55] Pronk S, Pall S, Schulz R, Larsson P, Bjelkmar P, Apostolov R, et al GROMACS 4.5: a high-throughput and highly parallel open source molecular simulation toolkit. Bioinformatics. 2013;29(7):845–54. doi: 10.1093/bioinformatics/btt055 23407358 10.1093/bioinformatics/btt055 PMC3605599

[56] Essmann U, Perera L, Berkowitz ML, Darden T, Lee H, Pedersen LG. A Smooth Particle Mesh Ewald Method. J Chem Phys. 1995;103(19):8577–93. doi: 10.1063/1.470117 ISI:A1995TE36400026

[57] Parrinello M, Rahman A. Polymorphic Transitions in Single-Crystals—a New Molecular-Dynamics Method. J Appl Phys. 1981;52(12):7182–90. ISI:A1981MT07800024

[58] Nose S. A Unified Formulation of the Constant Temperature Molecular-Dynamics Methods. J Chem Phys. 1984;81(1):511–9. ISI:A1984TA66100062

[59] Hoover WG. Canonical dynamics: Equilibrium phase-space distributions. Phys Rev A Gen Phys. 1985;31(3):1695–7. 9895674 10.1103/physreva.31.1695

