# Supplementary material for: Modeling of annexin A2—Membrane interactions by molecular dynamics simulations
Source: PLoS One. 2017 Sep 22;12(9):e0185440. doi: 10.1371/journal.pone.0185440 (PMC5609761; doi:10.1371/journal.pone.0185440)
Supplement: S1 Fig — Each pair shows the initial and final configurations of AnxA2 in respective system. The four repeats of AnxA2 are shown in different colors: repeats 1 (Ala35 –Lys104), 2 (Ala107 –Lys176), 3 (Asp187 –Ile263) and 4 (Lys266 –Cys335). The experimentally found Ca2+ binding Asp or Glu residues are shown in black. The Ca2+ ions are shown as red spheres. (PDF) [file pone.0185440.s001.pdf]

The initial and final configurations of AnxA2 in all 18 orientations are shown in S1 Fig.

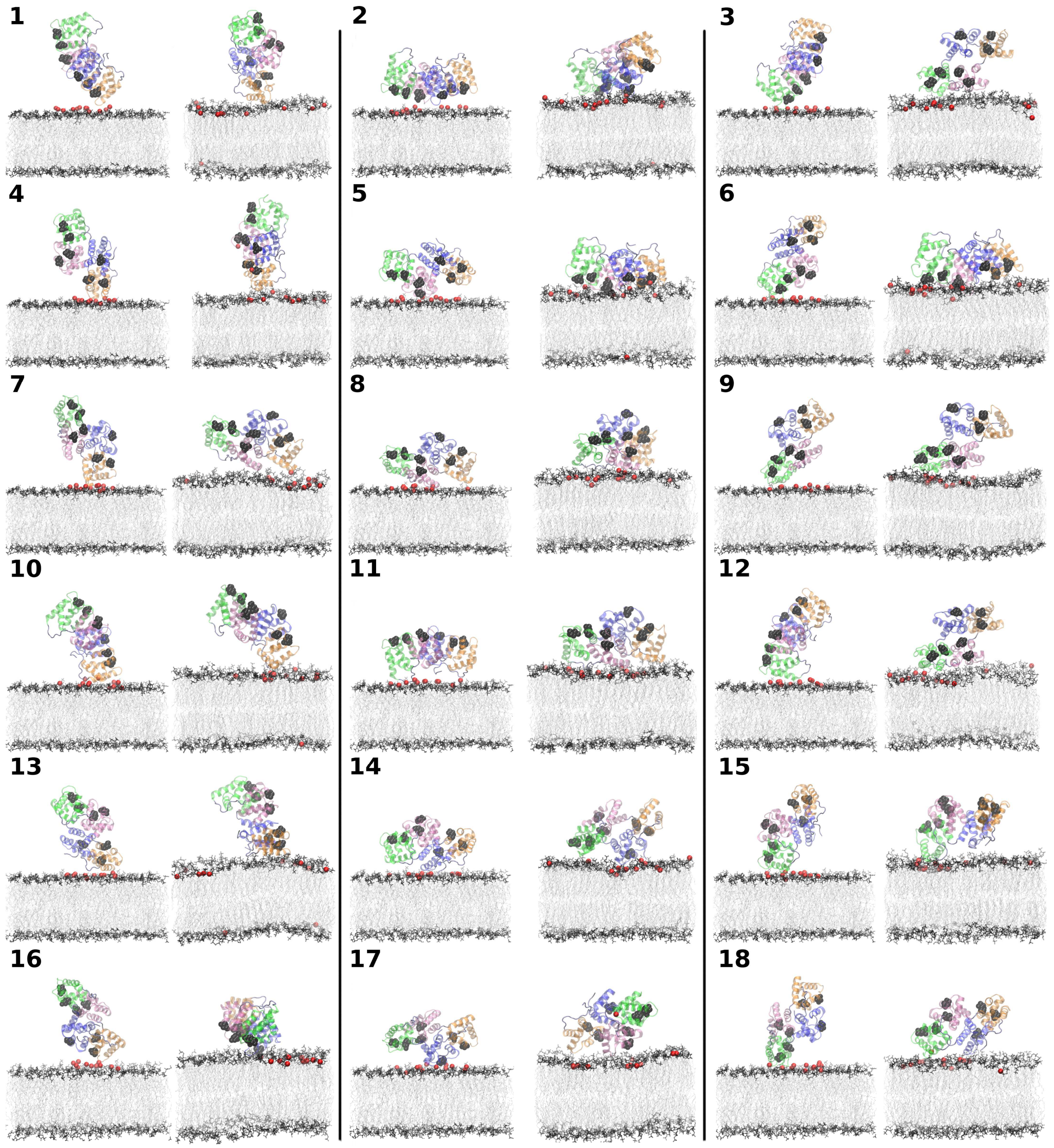

S1 Fig. The initial and final configurations of 18 systems. *Each pair shows the initial and final configurations of AnxA2 in respective system. The four repeats of AnxA2 are shown in different colors: repeats 1 (Ala35 – Lys104), 2 (Ala107 – Lys176), 3 (Asp187 – Ile263) and 4 (Lys266 – Cys335). The experimentally found Ca^{2+} binding Asp or Glu residues are shown in black. The Ca^{2+} ions are shown as red spheres.*
