# Supplementary material for: Modeling of annexin A2—Membrane interactions by molecular dynamics simulations
Source: PLoS One. 2017 Sep 22;12(9):e0185440. doi: 10.1371/journal.pone.0185440 (PMC5609761; doi:10.1371/journal.pone.0185440)
Supplement: S2 Fig — (A) System A. (B) System C. The upper and lower rows in each subplot represent membrane leaflets which are non-interacting and interacting with AnxA2, respectively. The first column represents the difference in the Z direction of the phosphor P atoms in all lipids relative to the average Z value in angstroms. The second column displays the total interaction energy of membrane-AnxA2 pair while the last column represent the total interaction energy of the membrane-Ca2+ pair. The energies in the second and third columns are in units of kcal/mol. The points in the X and Y planes reflect the coordinates of the P atoms of the lipids. (PDF) [file pone.0185440.s002.pdf]

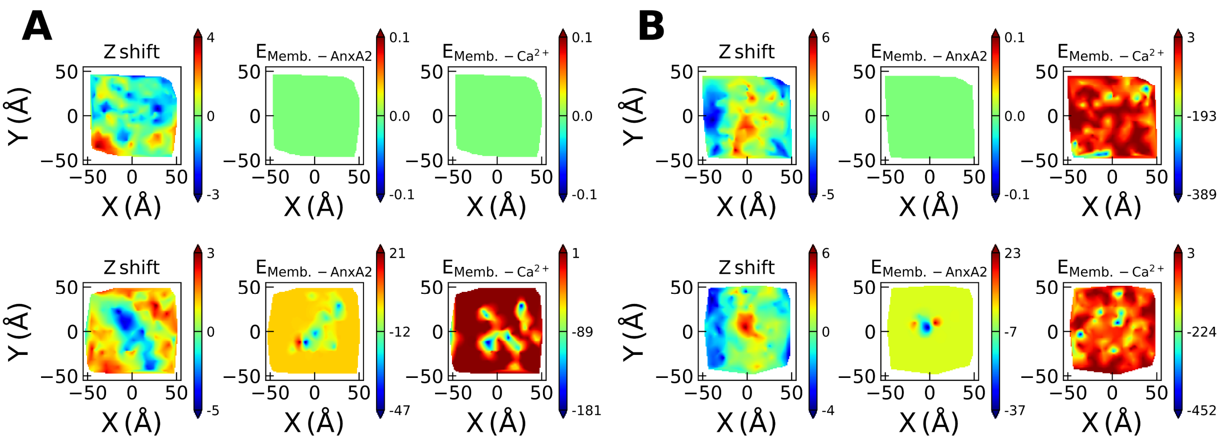

S2 Fig. Membrane bending with membrane-AnxA2 and membrane-Ca^{2+} interactions. *(A) System A. (B) System C. The upper and lower rows in each subplot represent membrane leaflets which are non-interacting and interacting with AnxA2, respectively. The first column represents the difference in the Z direction of the phosphor P atoms in all lipids relative to the average Z value in angstroms. The second column displays the total interaction energy of membrane-AnxA2 pair while the last column represent the total interaction energy of the membrane-Ca^{2+} pair. The energies in the second and third columns are in units of kcal/mol. The points in the X and Y planes reflect the coordinates of the P atoms of the lipids.*
