# Supplementary material for: Modeling of annexin A2—Membrane interactions by molecular dynamics simulations
Source: PLoS One. 2017 Sep 22;12(9):e0185440. doi: 10.1371/journal.pone.0185440 (PMC5609761; doi:10.1371/journal.pone.0185440)
Supplement: S3 Fig — (A) System A. (B) System C. In the upper and lower plots the horizontal axes represent membrane-AnxA2 and membrane-Ca2+ interaction energies, respectively. Shown are the average values for the last 20 ns of the 200 ns simulation. The PI(4,5)P2 and POPS lipids which significantly interacted with both AnxA2 as well as with Ca2+ ions are shown in yellow. (PDF) [file pone.0185440.s003.pdf]

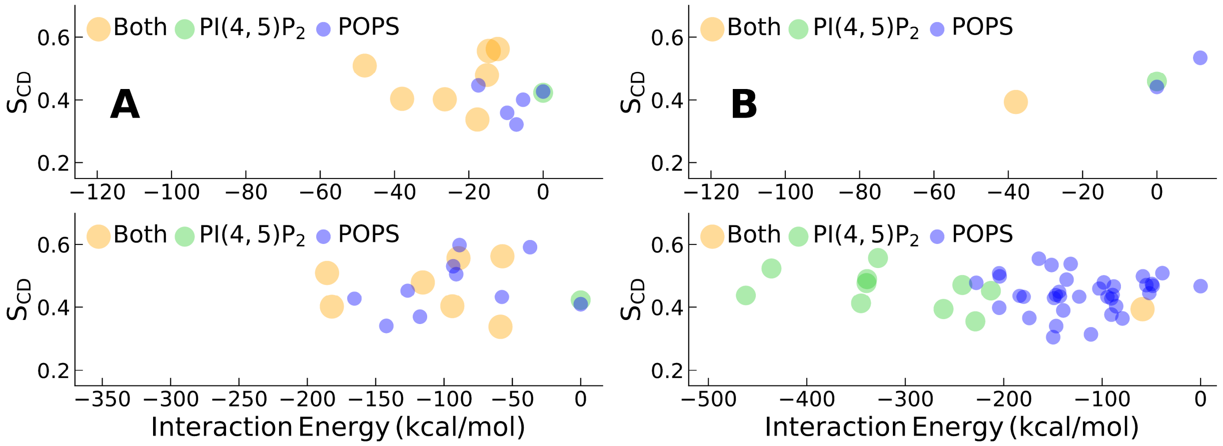


***S3 Fig. Lipid order parameters $S_{CD}$ as a function of membrane-AnxA2 and membrane-$Ca^{2+}$ interactions.*** *(A) System A. (B) System C. In the upper and lower plots the horizontal axes represent membrane-AnxA2 and membrane-$Ca^{2+}$ interaction energies, respectively. Shown are the average values for the last 20 ns of the 200 ns simulation. The PI(4,5)$P_2$ and POPS lipids which significantly interacted with both AnxA2 as well as with $Ca^{2+}$ ions are shown in yellow.*
