# Supplementary material for: Modeling of annexin A2—Membrane interactions by molecular dynamics simulations
Source: PLoS One. 2017 Sep 22;12(9):e0185440. doi: 10.1371/journal.pone.0185440 (PMC5609761; doi:10.1371/journal.pone.0185440)
Supplement: S1 Text — (PDF) [file pone.0185440.s004.pdf]

S1 Text

System C contained 150 Ca^{2+} ions and ~40000 water molecules. In that system 150 Ca^{2+} ions would correspond to 200 mM concentration. Experimental setups with a giant unilamellar vesicle (GUV) of 10 µm in diameter placed in an aqueous buffer with minimal size of 15 µm and 250 µM Ca^{2+} would correspond to the following estimated numbers:

Number of lipids in a GUV: ~ (5 – 10) x 10^8 (assumed average area per lipid 0.65 nm^2)
Number of water molecules: ~ 1.13 x 10^{14}
Number of Ca^{2+} ions (at concentration 250 µM): ~ 5 x 10^8

In such a sample, according to the above estimation the number of lipids and the number of calcium ions would be of the same order. Because of the high capacity of negatively charged lipids to absorb calcium ions, after some tenth of nanoseconds most of the Ca^{2+} ions should, presumably, be localized on the bilayer surface.

In this regard in our simulations for system C with 150 Ca^{2+} we coupled the number of calcium ions to the number of lipids rather than to the number of water molecules as the thickness of the water shell in the unit cell should not have much influence on the dynamics of Ca^{2+} ions other than the time required for calcium ions to be deposited on the leaflet of the bilayer.
